# Designation of highly efficient catalysts for one pot conversion of glycerol to lactic acid

**DOI:** 10.1038/srep29840

**Published:** 2016-07-19

**Authors:** Meilin Tao, Dan Zhang, Hongyu Guan, Guohui Huang, Xiaohong Wang

**Affiliations:** 1 Key Lab of Polyoxometalate Science of Ministry of Education, Northeast Normal University, Changchun 130024, P. R. China; 2 School of Biology, Northeast Normal University, Changchun 130024, People’s Republic of China

## Abstract

Production of lactic acid from glycerol is a cascade catalytic procedure using multifunctional catalysts combined with oxidative and acidic catalytic sites. Therefore, a series of silver-exchanged phosphomolybdic acid catalysts (Ag_x_H_3−x_PMo_12_O_40_, x = 1 ~ 3, abbreviated as Ag_x_PMo) was designed and applied in glycerol oxidation with O_2_ as an oxidant to produce lactic acid (LA) without adding any base. Among all, total silver exchanged phosphomolybdic acid (Ag_3_PMo) was found to be the most active one with LA selectivity of 93% at 99% conversion under mild conditions of 5 h at 60 °C. The exceptionally high efficiency was contributed to the generation of strong Lewis acid sites, enhanced redox potentials and water-tolerance. More importantly, Ag_3_PMo was tolerant in crude glycerol from biodiesel production. And the reaction mechanism was also discussed. Meanwhile, Ag_3_PMo acted as a heterogeneous catalyst for 12 recycles without loss of activity.

Cascade or tandem reactions are often carried out in one pot to maximize spatical and temporal productivity with mobilization of minimum resources^1^. From the “Green chemistry” point of view, cascade reactions can increase the economic competitiveness in the production of target compounds by reducing the isolation of synthetic intermediates and unnecessary purifications. The key point in cascade reaction is to create a new catalyst that is active to promote all the individual reactions involved in the cascade reactions^2^,^3^,^4^. By now, a series of cascade catalysts have been developed including those with acids and bases or redox sites as active centers. To fabricate such multifunctional catalysts, multi-components are usually needed comprising of noble metals Pt, Au, Pd or Ru and acids or bases through loading these metal nanoparticles on probable acid or base supports^5^. Because of the increasing interest for developing cascade reactions in catalysis, other new possible strategies to create higher numbers of catalytic centers are desirable and would open unforeseen avenues in catalysis. In addition, if there is a catalyst with more than two active sites such as acid, base and metal centers, it would be highly useful for direct conversion of bio-derived feedstock into fine chemicals^6^ to avoid forthcoming oil shortage and mitigate global warming. However, it is more difficult in the implementation of bio-derived resources into cascade processes because of rare existence of highly active moieties and numerous side-reactions. Therefore, it becomes clear that the use of new catalysts with more active sites is crucial for facing future challenges.

One of the most important cascade reactions in conversion of bio-based feedstock is the conversion of glycerol to green bulk chemicals^5^,^7^,^8^,^9^. Among these, lactic acid (LA) has gained significant interesting, which is considered as a top 12 biobased platform molecule and may serves as a useful precursor and platform chemical for producing polymers and green solvents^10^,^11^ with wide applications in packaging food, cosmetic, and pharmaceutical industry^12^,^13^. In generally, LA is mainly produced (approximately 95% of world production) from sugars and sugar alcohols via the fermentation route at strict temperature (<313 K) and pH 5–7 followed by purification, distillation and hydrolysis, which shows sustainability problems associated with the up-scaling of the current fermentation route and waste disposal^14^. Therefore, there is a persistent need to develop efficient methods for production of LA. An alternative production of LA from glycerol generally requires oxidation and then dehydration/rehydration steps to LA under reductive or inert conditions, or with oxidants or electrochemicals. By now, high-yield (80%) hydrothermal conversion of glycerol has been achieved in harsh alkaline conditions at a high temperature (573 K)^15^, which was not an economic process. Recently, a cascade reaction has been developed to selectively transform glycerol to LA under oxidative conditions using bio-and chemocatalysts, which is more logical^16^,^17^,^18^,^19^,^20^,^21^. Firstly, glycerol is selectively oxidized to dihydroxyacetone (DHA) and glyceraldehydes (GCA) in the presence of metal catalysts; secondly, GCA and DHA undergo dehydration to pyruvaldehyde (PAL); finally, PAL hydrated or benzilic acid rearranged to LA by base-catalysis. However, GCA and DHA are very prone to be further oxidated to the undesired by-product glyceric acid (GlyA) or tartronic acid. For the reported processes, LA was obtained via aerobic oxidation of glycerol catalyzed by noble-metal catalysts including Au-Pt/TiO_2_^16^ (85% selectivity at 30% conversion at 90 °C, 4 h), AuPt/CeO_2 _22 (80% selectivity at 99% conversion at 100 °C for 30 min), Pt_1_Ni_1_O_x_/TiO_2_^17^ (62.6% selectivity at 99.1% conversion at 90 °C for 2 h), and homogeneous Ir (I) bis-carbonyl complex^20^ (96% selectivity at 94% conversion at 130 °C for 24 h) under base. It can be concluded that the presence of base is critical to achieve a high conversion of glycerol under higher temperature or longer reaction time. However, the use of base led to the formation of salts instead of LA and required acidic treatment to isolate LA. Therefore, production of LA from glycerol oxidation under base-free conditions remains highly desirable. Unfortunately, only scare studies have succeeded in the chemocatalytic production of LA from glycerol without adding base under mild conditions^23^,^24^. Liu *et al*. reported that glycerol could be selectively converted to LA by Au-Pd/TiO_2_ combined with the Lewis acid AlCl_3_ with 100% conversion and 47.6% selectivity at 160 °C for 2 h^24^. However, dissociation of AlCl_3_ and poisoning by Cl^−^ remained issues in such a catalytic process. Fan *et al*. synthesized Pt/Sn-MFI as a heterogeneous catalyst giving 89.8% conversion of glycerol and 80.5% selectivity at 100 °C for 24 h with TOF 1.95^23^, but they faced the problem of leaching of Lewis acidic sites Sn from loadings. Most recently, M. Hara’s group reported combined Pt/TiO_2_ could catalyze one-pot conversion of glycerol to LA with 63% yield and 99% conversion at 150 °C for 18 h^25^. The above reports pointed that Lewis acids played an essential role for LA production, which accelerated the key step of benzilic acid rearrangement or an intramolecular 1.2-hydride shift^26^. Therefore, much effort has been done to develop active and durable combined catalytic systems of oxidation and heterogeneous Lewis acid catalysts for efficient cascade conversion of glycerol to LA in aqueous media.

Only recently, we found that a simple phosphomolybdic acid (H_3_PMo_12_O_40_) was highly efficient for cascade conversion of glycerol to LA in water^27^ giving 90% conversion and 79% yield of LA at 60 °C for 5 h. And we also for the first time found introduction of Lewis metals Al and Cr into H_3_PMo_12_O_40 _could enhance their redox ability, and hence to promote glycerol conversion directly to LA combined with Lewis acidity. These bifunctional catalysts - strong oxidative ability and Lewis acidity achieved production of LA with 90.5% selectivity at 93.7% conversion under mild reaction conditions^28^. As continuation of our research work, other Lewis metals could be introduced to H_3_PMo_12_O_40_ to evaluate their influence on redox ability and catalytic activity. Herein, Ag - exchanged heteropolyacids (HPAs) Ag_x_PMo with varying silver contents were designed to prepare and characterize for glycerol conversion under O_2_. Silver ions was used as Lewis centers being introduced to H_3_PW_12_O_40_, which showed higher efficient for glycerol esterification with acetic acid (AcA)^29^. And the Lewis acidic strength of Ag_x_H_3−x_PW_12_O_40_ (x = 1 ~ 3) was controlled by varying the contents of Ag. To the best of our knowledge, there is no report on Lewis center on redox HPAs apart from the only one report of Al or Cr in H_3_PMo_12_O_40_. Therefore, the systematical research for the influence of Lewis metals on redox ability and catalytic properties are essential not only for glycerol conversion, but also for other oxidative transformation using a cascade strategy based on multifunctional HPA catalysts. Also the pathway of production of LA from glycerol was checked in order to determine the influence of Ag centers on the conversion of glycerol and distribution of products as well. Meanwhile, conversion of crude glycerol from biodiesel production was done to determine the actual application of Ag_x_PMo.

## Experimental

### Material and reagent

All the chemicals were of AR grade, which were obtained commercially and used without further purification. H_3_PMo_12_O_40_ was synthesized according to the ref. 30.

### Instrument

Elemental analysis was carried out using a Leeman Plasma Spec (I) ICP-ES. IR spectroscopy (4000–500 cm^−1^) was recorded in KBr discs on a Nicolet Magna 560 IR spectrometer. X-ray diffraction (XRD) patterns of the sample were collected on a Japan Rigaku Dmax 2000 X-ray diffractometer with Cu Kα radiation (λ = 0.154178 nm). The measurements were obtained in the step of 0.04° with account time of 0.5 s and in the 2θ rang of 5–90 °. Raman spectroscopy was obtained on a Renishaw-UV-vis Raman System 1000 equipped with a CCD detector at room temperature. The air-cooled frequency doubled Nd-Yag laser operating at 532 nm was employed as the exciting source with a power of 30 MW. SEM micrographs were recorded on a scan electron microscope (XL30 ESEM FEG 25 kV). EDX spectra were obtained using 20 kV primary electron voltages to determine the composition of the samples. Water contact angle (CA) was carried out by the Contact Angle Meter using the droplet profile as a method. The CA was determined using a tangent placed at the intersection of the liquid and solid. A water droplet with a volume of 2 μL was dispensed by a piezo doser onto each sample disk. The redox potential were tested by cyclic voltammetry (CV) on a CS Corrtest electrochemical workstation equipped with graphite powder (SP) and liquid paraffin (4:1) as electrodes and saturated calomel as reference electrode. Electrochemical measurements were performed with 0.1 M sulfuric acid solution as the supporting electrolyte. The redox potential of Ag_x_PMo catalysts were tested using carbon paste electrode. A TOC analyzer (TOC-L CPH, Shimadzu, Japan) were used to monitor TOC values before and after reaction. The thermo gravimetric analyzer (TGA) and differential thermal analysis (DTA) were used to test the stability of catalysts.

Titration was used to evaluate the total acid content of the solids^31^. 0.05 g of Ag_x_PMo was suspended in 45 mL acetonitrile and then the mixture was stirred for 3 h. The density of acid sites in the catalysts was measured by titration with a solution of n-butylamine in acetonitrile (0.05 M) using the indicator anthraquinone (pKa = −8.2). The IR spectra of adsorbed pyridine (Py-IR) helped to measure the acid content and distinguish the properties of acid sites (Lewis or Brønsted). The samples were exposed to the pyridine vapor for 12 h under vacuum (10^−3^ Pa) at 60 °C. The quantification of acidity was calculated by Lambert–Beer equation (1):


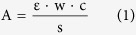


where A is the absorbance (area in cm^−1^), ε is the extinction coefficient (m^2^/mol), w is the sample weight (kg), c is the concentration of acid (mol/kg or mmol/g), and s is the sample disk area (m^2^), respectively. The amount of Brønsted and Lewis acid sites was estimated from the integrated area of the adsorption bands at 1540 and 1450 cm^−1^, respectively, using the extinction coefficient values based on the previous report^32^.

### Catalyst preparation

The Ag-exchanged H_3_PMo_12_O_40_ (abbreviated as HPMo) catalysts were synthesized by an ion-exchanged method, according to the procedure described previously^33^,^34^. Firstly, 9.125 g (0.005 M) of H_3_PMo_12_O_40_ was dissolved in 20 mL of deionized water at room temperature under vigorous stirring. Then, the appropriate amount of AgNO_3_ (0.005 M, 0.01 M and 0.015 M) aqueous solution was added dropwise to the former solution with continuous stirring. A yellow precipitate was formed immediately which was stirred for another 2 h. The large yellow precipitate was filtered and dried in nitrogen. From the TGA and DTA grams (Fig. S1), it can be seen that a decrease in weight up to 250 °C was corresponded to the loss of crystal water molecules in the material. So the yellow powder was calcined at 250 °C in static air for 4 h to loss their crystal water to obtain Ag_x_PMo with yield of 78.6%. It also can be seen that Ag_x_PMo were stable until 400 °C. The formation of Ag_x_PMo reaction undergoes based on the following equations:


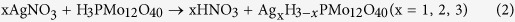


### Catalytic tests

Glycerol oxidation was performed in a high-pressure batch autoclave of stainless steel with a polytetrafluoroethylene inlet (10 mL). The autoclave was equipped with gas supply system and a magnetic stirrer. A catalyst was suspended in 5 mL aqueous glycerol, and the mixture was heated up to 60 °C. During the reaction, oxygen pressure was maintained at 10 bar. After the experiment, the reaction mixture was diluted 10 times with distilled water and analyzed by high performance liquid chromatography (HPLC) using a Shimadzu LC10A-VP chromatograph equipped with a SPB-10A Variable UV (210 nm) and a RID-10A R.I. detector. Prevail TM C18 column (4.6 mm × 250 mm) was used with a solution of H_2_SO_4_ (0.1% w/w) in H_2_O/acetonitrile (1/2 v/v) (1.0 mL min^−1^) as the eluent at 50 °C.

## Results and Discussion

### Catalyst characterization

**Figure 1:**
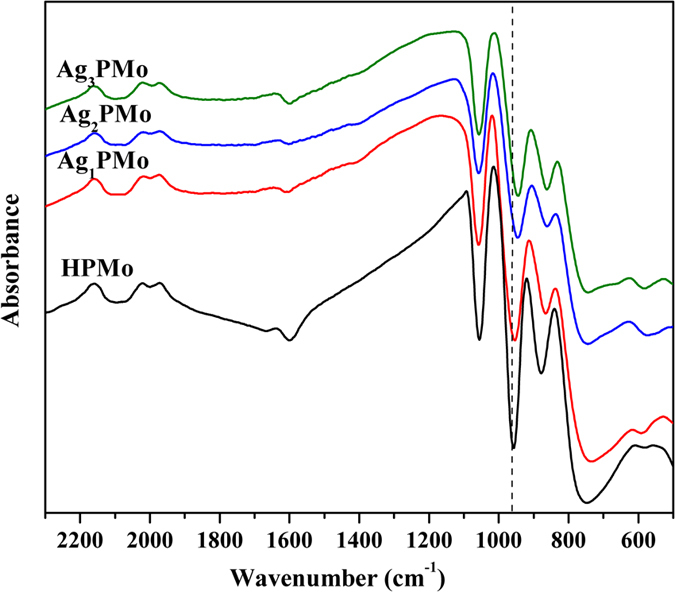
FTIR spectra of bulk HPMo and Ag-salt catalysts.

To thoroughly recognize the structure transformation induced by silver doping and morphologies of as-prepared Ag-exchanged HPMo catalysts, a combination of elemental analyses, FTIR, Raman, XRD, SEM-EDX, BET and TGA were employed. The elemental analyses of Ag_x_PMo were given in Table 1. The results gave the molar ratio of P: Mo = 1:12, showing the formation of dedecomolybdophosphates. The different Ag_x_PMo gave Ag: H as 1: 2, 2: 1 and 3: 0, indicating the formation of Ag_1_PMo, Ag_2_PMo and Ag_3_PMo, respectively. Infrared spectra are known to be an informative fingerprint of Keggin structure at molecular level^35^. FTIR spectra of parent HPMo and Ag_x_PMo were given in Fig. 1. In the case of parent HPMo, the characteristic bands of typical Keggin anion can be clearly observed as follows: 1060 cm^−1^ (νas P-O), 965 cm^−1^ (νas Mo = O), 882 cm^−1^ (νas Mo-O-Mo inter-octahedral), and 748 cm^−1^ (νas Mo-O-Mo intra-octahedral)^36^, respectively. All silver-exchanged HPMo catalysts exhibited these typical bands at similar frequencies, close to those of parent HPMo, indicating the whole retention of Keggin structure on the as-prepared Ag-salt catalysts. In addition, the shifts of νas Mo = O occurred from 965 cm^−1^ of HPMo to 942 cm^−1^ corresponding to Ag_1_PMo, Ag_2_PMo, and Ag_3_PMo, respectively. This was proposed to be the interaction between Ag and terminal oxygen of Mo = O leading to some blue shifts of the IR band of νas Mo = O, which was the main contribution to acceleration of electron transferring in oxidation^37^. Such blue shifts were correlative to the amount of Ag with the order of Ag_3_PMo (23 cm^−1^) >Ag_2_PMo (21 cm^−1^) >Ag_1_PMo (15 cm^−1^). This result also demonstrated that introduction of Lewis metal ions to HPAs could influence their electron transferring in oxidative reactions. The different redox potentials of as-prepared samples demonstrated this phenomenon (Table 1). As the increase of Ag amount, the redox potential increased from +0.63 V to +0.80 V corresponding to Ag_1_PMo, Ag_2_PMo and Ag_3_PMo, respectively.

**Figure 2:**
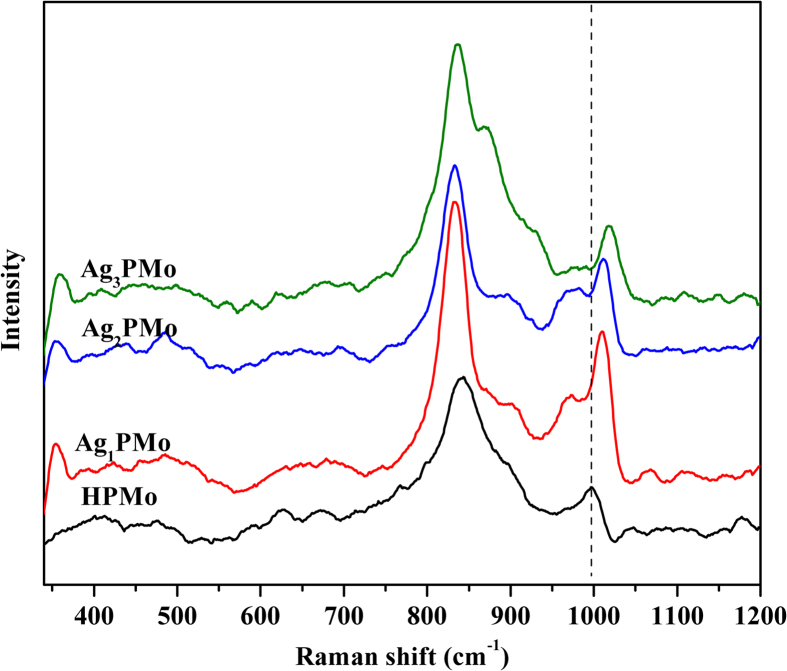
Raman spectra of bulk HPMo and Ag-salt catalysts.

Raman spectroscopy is uniquely suited for checking Keggin structure of HPAs to verify the presence and integrity of HPAs structure. Figure 2 showed Raman spectra of parent HPMo and its Ag-salt catalysts. It was identified and attributed Raman bands at 995 cm^−1^ and a shoulder at 965 cm^−1^ to the symmetric and asymmetric stretching modes of Mo = O^36^. The bands at 850 and 620 cm^−1^ were ascribed to the bending modes of Mo-O-Mo and O-P-O, respectively^36^. These bands were all clearly identified in the as-prepared silver-exchanged HPMo catalysts, suggesting that the Keggin structure was intact even after exchange of acidic protons with silver. Moreover, about 23 cm^−1^ shift also indicated the linkage between Ag and terminal oxygen.

**Figure 3:**
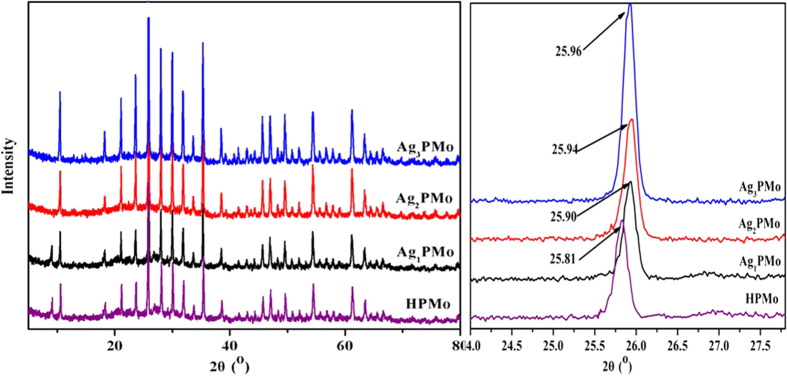
XRD patterns of bulk HPMo and Ag-salt catalysts.

Figure 3 gave the XRD patterns of parent HPMo and Ag-salt catalysts. The parent HPMo exhibited all of typical X-ray diffractograms of body-centered cubic secondary structure of Keggin anion, with characteristic diffraction peaks at 8.5, 10.3, 25.8 and 34.6° 38, respectively. Although the as-prepared Ag-salt samples exhibited very similar diffraction patterns as that of HPMo, a slight shift toward higher 2θ values was observed, suggesting the presence of Ag_x_PMo. A detailed study of the predominant 25.0–27.0 ° reflections confirmed this effect, showing the continuous shift toward lower lattice parameters with the increase in silver content, as reported previously^39^. It is speculated that silver-exchanged HPMo catalysts possess the same symmetry as parent HPMo but with a contracted unit cell. This contraction of unit cell can be explained by the exchange of protons present in the secondary Keggin structure in the form of hydronium ions H_3_O^+^ for hydrated silver cation(Fig. S2). The same behavior was observed by Borgh’ese *et al*. in the case of silver-exchanged silicotungstic acids catalysts^33^,^34^. Accordingly, our XRD patterns revealed the successful incorporation of Ag^+^ into HPMo anion with the remaining of Keggin structure.

**Figure 4:**
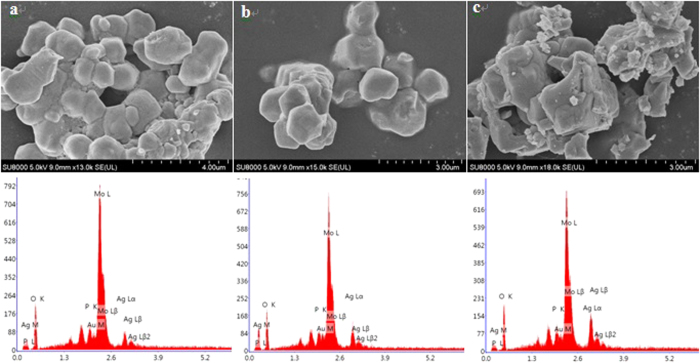
SEM micrographs and EDX of (a) Ag1PMo, (b) Ag2PMo, and (c) Ag3PMo catalysts.

To gain further insights, SEM was conducted to directly image the as-prepared catalysts. A representative selection of SEM images of Ag_1_PMo, Ag_2_PMo and Ag_3_PMo samples were presented in Fig. 4. It can be found that these as-synthesized materials displayed well-shaped crystalline particles, particularly for the fully substituted Ag_3_PMo. With the increase in Ag content, the size of crystalline particles evidently grew, which was because of the replacement of H^+^ (with a smaller ionic radius) by Ag^+^ (with a bigger ionic radius) as the demonstration in Fig. S2. Furthermore, EDX was examined to determine the elemental compositions of these samples.

From Fig. S3, it can be seen that Ag_x_PMo formed mesopore structure and the pore sizes were 4.42, 4.86, and 4.93 nm corresponding to Ag_1_PMo, Ag_2_PMo and Ag_3_PMo, respectively. Fig. S4 also displayed that the catalysts gathered to form micropores somewhile.

**Figure 5:**
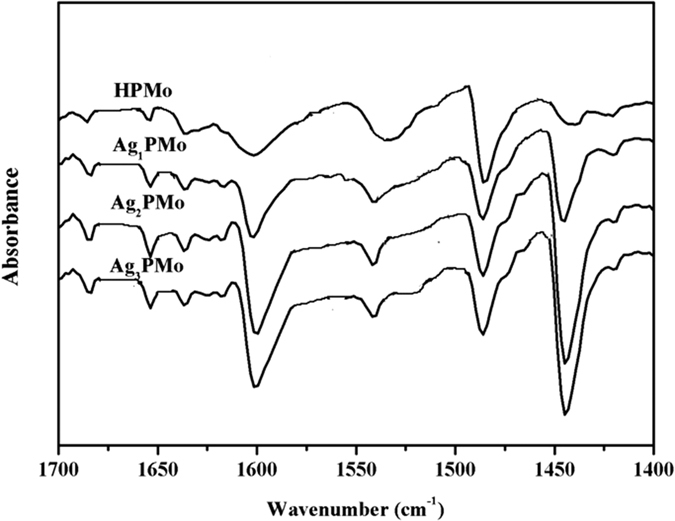
FTIR spectra of pyridine adsorption of bulk HPMo and Ag-salt catalysts.

For the cascade conversion of glycerol to LA, both the surface acid density and the nature of acid sites play the key roles in determining the catalytic performance. Therefore, FT-IR spectra of pyridine absorption were recorded to probe accessible surface acid sites, which is a powerful tool for identifying the nature of acid sites. As shown in Fig. 5, all the samples presented typical bands corresponding to strong Brønsted acid bound pyridine, at around 1540 and 1639 cm^−1 ^40. The bands at around 1450 and 1610 cm^−1^ were assigned to the coordinated pyridine adsorbed on Lewis acid sites, while the band at 1489 cm^−1^ was originated from the combination of pyridine on both Brønsted and Lewis acid sites^41^. The concentrations of Brønsted acid and Lewis acid sites were obtained from the bands at 1540 and 1450 cm^−1^ based on Lambert-Beer equation, and the calculated results were listed in Table 2. In comparison with pure Brønsted acid of parent HPMo, Lewis acid sites emerged as a result of the exchange of Ag with protons of HPMo, originating from the coordinately unsaturated Ag species in the catalyst. Among them, the Ag_1_PMo presented the maximum acidity, even more than the parent HPMo, owing to the enhanced Lewis acid sites. In comparison with the fully Ag-exchanged HPMo sample, partially Ag substituted HPMo catalyst increased available acid sites, due to the presence of residual protons capable of mobility and inducing new strong acid sites^35^. Similar behavior has been reported in the cases of Cs^+^ and Sn-exchanged HPW catalysts^35^,^42^. It was interesting to find that the fully Ag-exchanged HPMo sample presented 0.08 mmol/g Brønsted acids, which may originate from the adsorbed surface OH groups or even remaining trace protons. Totally, the molar ratio between Brønsted acid and Lewis acid were 0.86, 0.87, and 0.96 corresponding to Ag_1_PMo, Ag_2_PMo, and Ag_3_PMo, respectively.

**Figure 6:**
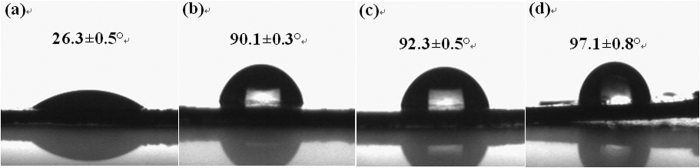
The CA of the two surfaces of HPMo (a), Ag_1_PMo (b), Ag_2_PMo (c), and Ag_3_PMo (d).

Measurement of water contact angle is the most common method for determining hydrophobic property of materials. From Fig. 6, it can be seen that the water contact angle values of Ag_x_PMo increased as the growth of Ag number as 90.1 ± 0.3, 92.3 ± 0.5 and 97.1 ± 0.8 ° corresponding to Ag_1_PMo, Ag_2_PMo and Ag_3_PMo, respectively, indicating that they all exhibited hydrophobic property compared to their parent H_3_PMo_12_O_40_, which gave a water contact angle of only 26.3 ± 0.5 °. The hydrophobic property of Ag_x_PMo was resulted from the introduction of Ag ion to H_3_PMo_12_O_40_ and the decrease of proton numbers.

### Catalytic activity of various catalysts and reaction pathways

The conversion of glycerol to LA reportedly is atom-economic but requires both redox conversion and dehydration/rehydration steps in the presence of a base or Lewis acidic catalysts^24^, which needs cascade catalysts. Side reactions, such as C–C cleavage, over-oxidation, and over-reduction, must be minimized to obtain the highest yield of LA, which is difficult to be controlled with catalysts operated under harsh reaction conditions. Therefore, to escape over-oxidation, middle oxidative ability of a catalyst is essential for glycerol to LA. In order to evaluate the effect of redox property on glycerol conversion, a number of phosphorus HPAs were selected including HPMo, H_4_SiMo_12_O_40_, H_4_SiW_12_O_40_, H_3_PW_12_O_40_, H_5_PMo_10_V_2_O_40_, K_3_PMo_12_O_40_, Ag_1_PMo, Ag_2_PMo, and Ag_3_PMo (Table 3). For free proton HPAs, the conversion of glycerol was in the following order: H_5_PMo_10_V_2_O_40_ (90%) >H_3_PMo_12_O_40_ (83%) >H_4_SiMo_12_O_40_ (75%) >H_4_SiW_12_O_40_ (68%) >H_3_PW_12_O_40_ (65%). H_5_PMo_10_V_2_O_40_ exhibited the highest activity, whereas H_3_PW_12_O_40_ was least active. The order of redox potentials were measured as H_5_PMo_10_V_2_O_40_ (+0.93 V) >H_3_PMo_12_O_40_ (+0.22 V) >H_4_SiMo_12_O_40_ (−0.39 V) >H_4_SiW_12_O_40_ (−0.46 V) >H_3_PW_12_O_40_ (−0.69 V). It is well known that for conversion of glycerol to LA, the first step is to oxidize glycerol to DHA or GCA requiring stronger oxidative catalysts. H_5_PMo_10_V_2_O_40_ exhibited the highest redox ability resulting in highest conversion of glycerol. H_4_SiW_12_O_40_ and H_3_PW_12_O_40_ exhibited too low oxidation properties to oxidize glycerol. As for the yields of LA, the range was opposite to active order as H_3_PMo_12_O_40_ (60%) >H_4_SiMo_12_O_40_ (50%) >H_4_SiW_12_O_40_ (41%) >H_3_PW_12_O_40_ (38%) >H_5_PMo_10_V_2_O_40_ (33%), showing that the catalyst with too high redox potential could not give the highest yield of LA because of the further oxidation. H_5_PMo_10_V_2_O_40_ gave much higher amount of GlyA and over oxidized product AcA due to its high oxidative ability. In order to obtain highest efficiency for glycerol to LA, HPMo was optimized as an available catalyst with middle oxidation-reduction potential. However, H_3_PMo_12_O_40_ is a homogeneous catalyst in water system that faces the problem of separation and unsatisfactory glycerol conversion.

**Figure 7:**
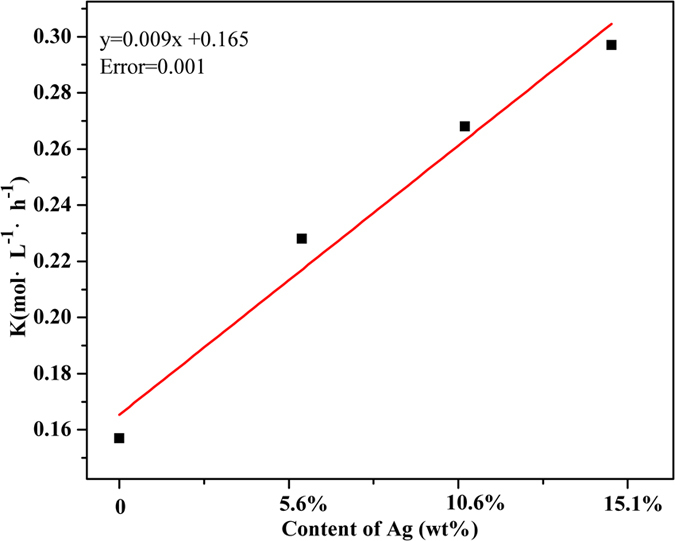
Influence of Ag^+^ concentration on glycerol oxygenation by AgPMo catalysts. Conditions: 5 mL, 1.1 M of glycerol, 2.3 × 10^−5 ^mol of AgPMo, 5 bar O_2_, 800 rpm, 2 h.

For phosphomolybdates, Ag_3_PMo exhibited the highest activity with glycerol conversion up to 89% under reaction conditions as 5 mL of glycerol solution (1.1 M), 2.3 × 10^−5^ mol of catalyst, using 5 bar O_2_ at 60 °C for 5 h. The conversion range was Ag_3_PMo >Ag_2_PMo >Ag_1_PMo >HPMo >K_3_PMo_12_O_40_. As-prepared Ag-salt catalysts presented an enhanced activity for glycerol oxidation compared to their parent and heterogeneous K_3_PMo_12_O_40_. K ion is a metal ion without any Lewis acidity and did not have positive effect for acceleration the reaction rate. Apparently, the greatly improved catalytic activity of HPMo by adding Lewis metal ions can be attributed to the synergistic effect between the PMo_12_O_40_^3+^ and the Lewis acid in oxygenation. Notably, the added metal ions with Lewis acidity having a higher positive charge demonstrated a better efficiency in promoting the catalytic efficiency of PMo_12_O_40_^3+^. This effect was attributed to the introduction of Lewis metal ions Ag resulting in shifts 0.43, 0.55, 0.60 V to the positive direction (Fig. S5) corresponding to Ag_1_, Ag_2_ and Ag_3_, respectively, as compared to that of K_3_PMo_12_O_40_ (0.15 V). Moreover, as demonstrated in Fig. 7, increasing the ratio of Lewis acid/PMo_12_O_40_^3+^ would further accelerate the oxygenation rate of glycerol oxidation. In absence of Lewis metal, the *k* (rate constant) value for glycerol oxidation by K_3_PMo_12_O_40_ was 0.157 mol·L^−1^·h^−1^, while they were 0.228, 0.268, 0.297 mol·L^−1^·h^−1^ in the presence of Ag_1_, Ag_2_ and Ag_3_, representing 1.5, 1.7, 2.0 fold oxidative rate increase, respectively. For LA yields, Ag_x_PMo present increasing LA yields from 39% over H_3_PMo_12_O_40_ to 64, 68 and 72%, respectively. In addition, the TOF (TOF = (concentration of formed LA, mol L^−1^)/((amount of HPA, mol L^−1^) × (reaction time, h)) of Ag_x_PMo was given with the order of Ag_3_PMo (35 h^−1^) >Ag_2_PMo (33 h^−1^) ~Ag_1_PMo (32 h^−1^) >HPMo (30 h^−1^). Meanwhile, the total organic carbon (TOC) before and after glycerol oxidation were 39.0 mg/mL and 38.6 mg/mL, respectively. It can be concluded that no gases were formed during the reaction, showing that glycerol was converted into LA with high selectivity. The enhanced LA yields over Ag_x_PMo were also contributed to slow further oxidation of LA to AcA (Table 3, entry 19–22) in comparison to H_3_PMo_12_O_40_.

**Figure 8:**
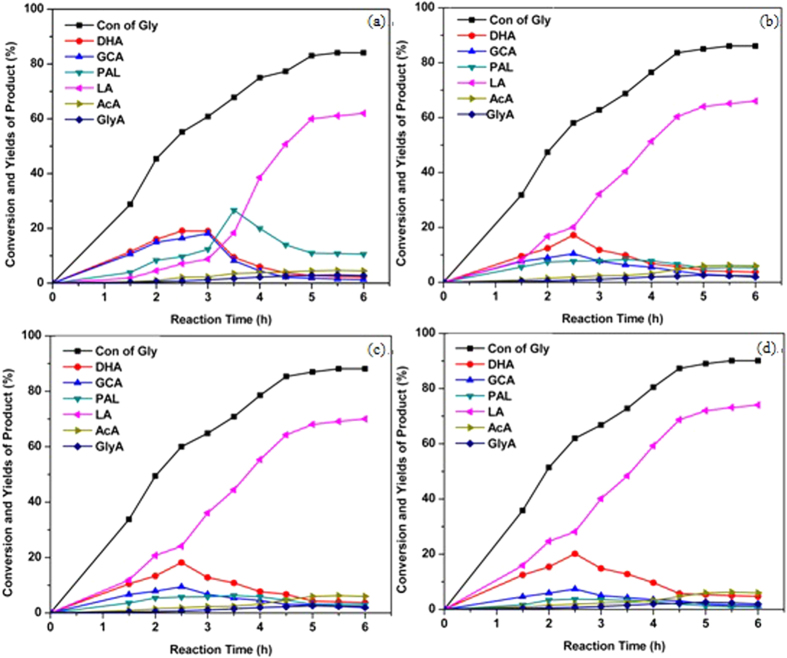
Time course of glycerol and the products. (**a**) HPMo, (**b**) Ag_1_PMo, (**c**) Ag_2_PMo, (**d**) Ag_3_PMo. Reaction conditions: 5 mL, 1.1 M of glycerol, 2.3 × 10^−5^ mol of MPMo, 5 bar O_2_, 800 rpm.

Figure 8 gave glycerol conversion and the representative product distributions verse reaction time catalyzed by HPMo and Ag_x_PMo. It can be found that (1) as expected, the glycerol conversion increased with the reaction time. And the predominant product of glycerol oxidation catalyzed by HPAs was LA accompanied by DHA, GCA, PAL, GlyA, and AcA. Therefore, it can be proposed that oxidation of glycerol to LA undergoes three steps: firstly, glycerol is oxidized to DHA and GCA; then, dehydration of DHA and GCA gives rise to PAL; and the last step is the benzylic acid rearrangement-like transformation of PAL to LA. (2) For HPMo (Fig. 8a), the formation of GCA and DHA was in equilibrium suggesting that Brønsted acid had no selectivity to the intermediate products. But, oxidation of glycerol over Ag_x_PMo (Fig. 8b–d) produced much more DHA than GCA, while increasing the number of Ag gave rise to much more DHA. It is plausible to believe that HPAs with Lewis acidity might be more selective for catalyzing the oxidation of secondary hydroxyl group of glycerol to yield DHA. This is consistent with our observation that DHA led to higher yields of LA than GCA when used as starting materials (Table 3, entry11–18). And much more Ag in HPAs gave rise to less yield of GCA. (3) The formation of GCA and DHA reached maximum value at a reaction time of 3.5 h then further increasing reaction time did not increase their summed yields over HPMo, while it needed 2.5 h over Ag_x_PMo. This might be attributed to the different redox potentials for HPMo and Ag_x_PMo as above discussion. (4) At 3.5 h, the summed yield for the intermediates DHA+ GCA was 16.1%, whereas the maximum yield to intermediates did not exceed 27.8% in the presence of Ag_x_PMo at 2.5 h. HPMo demonstrated lower activity in dehydration of DHA and GCA intermediates to PAL compared to Ag_x_PMo. Therefore, the fast transformation of DHA and GCA to LA catalyzed by Ag_x_PMo might further impel the glycerol conversion. Therefore, the formation of PAL mainly depended on Lewis acidity^43^,^44^. Another proposed reason was due to the relative hydrophobic surface of the Ag_x_PMo compared to hydrophilic surface of HPMo. Insoluble Ag_x_PMo salts are highly water-tolerant and capable of rapidly releasing of generated water from their secondary structure to promote the dehydration of DHA to PAL. The more water-tolerance is, the rapid speed of generation of LA is. Therefore, Ag_3_PMo presented the highest yield of LA under the same reaction conditions. (5) The yields of PAL were lower but the yields of LA were higher catalyzed by Ag_x_PMo than by HPMo. Thus, Ag cation with a Lewis acid site had a positive effect on activity and chemoselectivity and played a key role in the reaction pathways leading to LA. In order to test this hypothesis, the compared performance of HPMo for the conversion of DHA to LA was evaluated. From Table 3 (entry 11–18), it can be seen that dehydration rates of DHA or GCA catalyzed by HPMo were slower then by Ag_x_PMo. (6) The influence of Ag amount on the conversion of DHA to LA (entry 11–14) was that higher amount of Ag gave higher efficiency from DHA to LA. This further demonstrated the influence of Lewis sites on glycerol conversion to LA. (7) The higher yield of LA over Ag_x_PMo was contributed to the less further oxidation to pyruvic acid or AcA (Table 3, entry 19–22). The reason why undesired cascade oxidation of LA was hindered was their unique surface characteristics — the hydrophobic surface. As soon as LA formed, the hydrophobic Ag_x_PMo expelled LA molecules from their catalytic sites diffusing to the liquid phase and further oxidation was avoided. H_3_PMo_12_O_40_ has no such property and LA molecules were prone to be oxidized.

**Figure 9:**
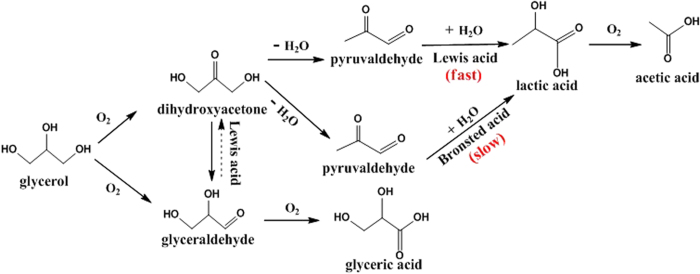
Proposed tandem reaction pathways for the selective oxidation of glycerol to lactic acid over the Ag_x_PMo catalysts.

Based on the above results, we proposed the effect of different catalytic sites on each pathway leading to LA from glycerol (Fig. 9). It can be concluded that optimized redox PMo_12_O_40_^3−^ center is mainly responsible for glycerol oxidation to DHA and GCA, while Lewis metal Ag could enhance the redox potentials for HPMo through the interaction between oxygen of PMo_12_O_40_^3−^ and Ag^+^, hence to promote the oxidation of glycerol. Brønsted acid sites favor for generation equilibrium DHA and GCA, while Lewis acid sites are responsible for the formation of DHA which could be dehydrated to PAL much easily. Then PAL converts into LA faster catalyzed by Lewis acid sites rather than by Brønsted acid sites. Therefore, the highest yield of LA was obtained though the synergistic catalysis between redox PMo_12_O_40_^3−^ and Lewis center Ag^+^.

The significant activity of Ag_x_PMo was also attributed to their absorption of glycerol determined by the IR spectra of as-prepared catalysts adsorption of glycerol (Fig. S6). It can be seen that the peaks at 3258, 1360 and 1108 cm^−1^ were attributed to the stretching vibrations of OH and C=O from glycerol, respectively, showing that some glycerol molecules were absorbed by Ag_x_PMo catalysts. Meanwhile, the strength of these peaks increased as the increasing of Ag amounts, which was in agreement with the activities (conversion of glycerol and yield to LA) of Ag_x_PMo with various number of Ag.

### Optimization of reaction conditions catalyzed by Ag_3_PMo

The above results revealed that the best glycerol conversion to LA was achieved by Ag_3_PMo. In order to optimize LA production, the different reaction conditions, including temperature, reagent concentrations, amount of catalyst, and reaction time were determined (Fig. S7) under Ag_3_PMo catalyzing. As observed, increasing the temperature from 40 to 60 °C led to an increase of conversion from 68 to 99% and also increases of LA selectivity from 64 to 93% at 5 h (Fig. S5a). Ag_3_PMo was active even at lower temperature of 40 °C, while the best efficiency was achieved as 98% yield at 100% conversion as increasing reaction time to 24 h. Further enhancement of temperature to 70 °C or 80 °C did not improve the activity significantly only giving decreasing selectivity. To the best of our knowledge, it is the highest yield of LA achieved under such lower temperature by now.

The conversion of glycerol and the selectivity to LA increased considerably as enhancement of catalyst usage from 0.007 to 0.023 mmol, achieving the highest of 99% conversion and 93% selectivity using 0.023 mmol of catalyst (Fig. S7b). Further increasing the amount of Ag_3_PMo to 0.039 mmol did not give significant increasing trend for conversion but a little decreasing of LA yield. Thus, the oxidation of glycerol was performed at this Ag_3_PMo usage of 0.023 mmol as the molar ratio of glycerol to Ag_3_PMo as 239:1, which was comparable to noble metal Pt. And also Ag_3_PMo was most active species among solid catalysts even at catalyst loadings as low as 0.02 mmol.

Figure S7c showed the effect of reaction time on glycerol conversion. It suggested that the conversion of glycerol and selectivity to LA increased as longing the time. While after 5 h, there was little change either on conversion or selectivity.

In the investigation of the influence of reagent concentrations (Fig. S7d), the conversion initially increased as the selectivity of LA increased and then decreased as the amount of glycerol increased. This result could be attributed to the increased dihydroxyacetone yield as the amount of glycerol increased, and the dihydroxyacetone required more time to transform to LA. Most importantly, the highest selectivity to LA (93%) was by Ag_3_PMo with 1.1 M of glycerol at 60 °C for 5 h.

It is interesting to find that Ag_3_PMo could successfully catalyze neat glycerol convert to LA under the optimized reaction conditions (5 mL neat glycerol, 0.023 mmol of Ag_3_PMo, 10 bar O_2_, 800 rpm, 60 °C) with 66% selectivity at 82% conversion for 24 h. This is a very useful result to improve the LA productivities compared to those using low glycerol concentrations.

From Fig. S7e, the conversion of glycerol and yield to LA increased dramatically as increasing the pressure of oxygen, while the selectivity to LA reached 93% with the glycerol conversion of 99% under 10 bar of oxygen. This was attributed to that more oxygen could accelerate the transform from glycerol to DHA in the first step. In view of the economics, air was used to replace oxygen in glycerol oxidation. To our delight, Ag_3_PMo showed a decreased but still very satisfactory activity in glycerol conversion into LA with 54% yield to LA at glycerol oxidation of 68% within 20 h.

### Reusability of Ag_3_PMo catalyst

**Figure 10:**
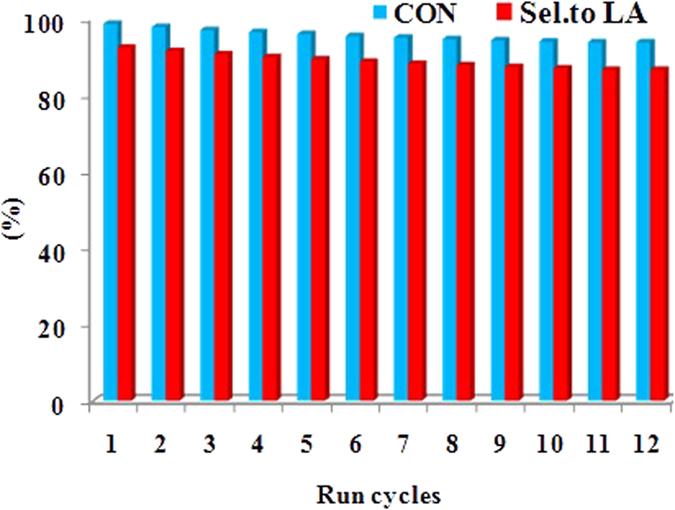
Reusability test catalyzed by Ag_3_PMo in oxidation of glycerol. Reaction conditions: 2.3 × 10^−5 ^mol of catalyst, 1.1 M of glycerol (5 mL), 10 bar, 60 °C, 5 h.

To assess the reusability of Ag_x_PMo catalyst for glycerol oxidation, the spent catalyst was separated from the reaction system by centrifugation after the completion of each run. The spent sample was washed with water, dried at 60 °C overnight prior to its reuse for further reaction cycle under the identical reaction conditions. As illustrated in Fig. 10, Ag_3_PMo catalyst exhibited similar activity for glycerol oxidation until 12 reaction cycles without significant loss of its activity. The conversion of glycerol and the yield to LA only decreased 4.8% and 8.9% after twelve cycles compared to the fresh one, respectively. And the total loss of Ag_3_PMo was about 5.2% of its initial amount.

In order to determine whether Ag_3_PMo leached into mixture or not, the mixture after the reaction was studied by UV-Vis spectroscopy (Fig. S8). It can be seen that there were no characteristic peaks of Ag_3_PMo in the range of 200–400 nm, showing no leaching of Ag_3_PMo into the reaction mixture. To further determine the leaching of Ag_3_PMo, the catalyst was separated after reacting for 2 h (51% of glycerol conversion) and was allowed to react further for over 2 h at the same conditions. The result showed that the conversion of glycerol was only 56.5%, which meant that Ag_3_PMo acted as a heterogeneous catalyst without leaching into the reaction mixture during the reaction. The loss of Ag_3_PMo was attributed to the operation.

The above results have demonstrated that the Ag_3_PMo catalyst is rather durable and holds the potential for practical applications. The excellent reusability of Ag_3_PMo was contributed, on the other hand, to its stability during the oxidation reaction, which was determined by IR, XRD, Raman and SEM spectroscopy (Figs S9–12). It can be seen that the spent catalyst displayed similar characteristic bands as the fresh one, revealing that the Keggin structure of Ag_x_PMo catalysts remained intact during the reaction. Therefore, Ag_x_PMo was rather stable and no leaching under the reaction conditions during the repetitive runs.

### Conversion of crude glycerol

Glycerol is a side-product of biodiesel industry with ca. 3 × 10^6^ tons in 2014. Impurity of crude glycerol in quantities mikes it difficult to use in most applications^45^. Production of LA from crude glycerol is of great interesting and economics, which provides an alternative without any cost of the purification. By now, direct conversion of crude glycerol was via fermentative processes^46^, but only a limited number of microorganisms could be tolerant to complex impurities in the crude mixture. Chemical conversion of crude glycerol is to product hydrogen and syngas, direct transformation into valued-added products is lacking^44^. There was only one report on chemical transformation of crude glycerol to LA using homogeneous Ir complex as catalyst under alkaline condition with 98% conversion and 96% LA selectivity at 130 °C for 24 h^20^.

Here used crude glycerol was obtained from biodiesel production without any purification comprising 71 wt% of glycerol, 28 wt % of methanol and other minor organic admixtures. Ag_3_PMo was found to be with high efficiency in transformation of crude glycerol with almost the same conversion (98%) and selectivity (92%) under mild conditions (5 mL, 1 M of crude glycerol, 0.023 mmol of Ag_3_PMo, 10 bar O_2_, 800 rpm, 60 °C, 5 h). This result suggests Ag_3_PMo to be a methanol-tolerant catalyst capable of converting crude glycerol. And the esterification of LA with methanol was hindered.

## Conclusion

Silver-exchanged phosphomolybdic acid catalysts Ag_x_PMo_12_O_40_ (x = 1, 2, 3) was prepared and evaluated for conversion of glycerol directly to lactic acid for the first time. It was found that Lewis metal Ag and its amounts could enhance the redox potentials for H_3_PMo_12_O_40_ to be suitable for oxidation of glycerol to intermediates DHA rather than GCA, while DHA is rather easily dehydrated to PAL than GCA. And then Ag, Lewis center site, favored for further dehydration reaction pathway to LA. Suitable redox potentials, Lewis center sites, and hydrophobic properties render their highly conversion and selectivity. Therefore, a remarkable 93% selectivity to lactic acid at 99% conversion was reached and the undesired cascade oxidation of lactic acid to further AcA was hindered over Ag_3_PMo. More importantly, production of LA from glycerol was achieved under mild conditions (60 °C, 5 h), catalyst concentration as low as 0.023 mmol, without adding solvent or alkaline, reusability of Ag_3_PMo, and tolerant to crude feedstocks. It fulfills the “green chemistry” concept. To economics and environmental benign, Ag_3_PMo as a catalyst for LA synthesis can be highlighted to convert crude glycerol (LA yield 92% under optimized conditions) as well as neat glycerol (LA yield 54% under optimized conditions for 24 h). Stability and no leaching of Ag_3_PMo during twelve runs showed that it is rather durable and holds the potential for practical applications.

This study provides a new alternative chemical transformation of glycerol to value-added chemicals under base free with no alkali. This also provides guidance for HPA application in cascade reactions for other biomass-related transformations or organic synthesis.

## Additional Information

**How to cite this article**: Tao, M. *et al*. Designation of highly efficient catalysts for one pot conversion of glycerol to lactic acid. *Sci. Rep.* **6**, 29840; doi: 10.1038/srep29840 (2016).

## Supplementary Material

Supplementary Information

## Figures and Tables

**Table 1:** Elementary results (calculated values in parenthesis)/wt% and redox potential of different catalysts.

Catalysts	H	P	Mo	Ag	redox potential(V)
HPMo	0.3 (0. 2)	1.3 (1.7)	60.0 (63.0)	—	+0.20
Ag_1_PMo	0. 2 (0. 1)	1.2 (1.7)	64.2 (59.7)	5.5 (5.6)	+0.63
Ag_2_PMo	0. 1 (0. 1)	2.2 (1.5)	54.3 (56.5)	10.2 (10.6)	+0.75
Ag_3_PMo	—	0. 9 (1.4)	48.8 (53.7)	14.4 (15.1)	+0.80

**Table 2:** Amount of acid sites on bulk HPMo and Ag-salt catalysts determined by FT-IR spectra of pyridine absorption.

Catalysts	Brønsted acidity(mmol/g)	Lewis acidity(mmol/g)	Total acidity(mmol/g)
HPMo	1.01	0.03	1.04
Ag_1_PMo	0.54	0.69	1.23
Ag_2_PMo	0.25	0.76	1.01
Ag_3_PMo	0.08	0.86	0.94

**Table 3:** Oxidation of glycerol in the presence of various HPA catalysts.

Entry	Catalyst	Substrate	TOF, h^−1^^b^	CON, %^a^	Yield LA, %	Selectivity (%)					
						DHA	GCA	PA	LA	GlyA	AcA
1	Blank	Glycerol	0	7	0	58	42	0	0	0	0
2	H_5_PMo_10_V_2_O_40_	Glycerol	16	90	33	6	5	4	37	23	25
3	H_4_SiMo_12_O_40_	Glycerol	24	75	50	8	7	10	67	4	4
4	H_4_SiW_12_O_40_	Glycerol	20	68	41	13	11	9	60	3	4
5	H_3_PMo_12_O_40_	Glycerol	30	83	60	3	3	13	72	4	5
6	H_3_PW_12_O_40_	Glycerol	19	65	38	19	15	6	58	1	1
7	K_3_PMo_12_O_40_	Glycerol	19	70	39	12	11	12	56	4	5
8	Ag_3_PMo_12_O_40_	Glycerol	35	89	72	6	2	1	81	3	7
9	Ag_2_HPMo_12_O_40_	Glycerol	33	87	68	5	3	4	78	3	7
10	Ag_1_H_2_PMo_12_O_40_	Glycerol	32	85	64	5	3	6	75	4	7
11	Ag_3_PMo_12_O_40_^c^	DHA	46	76	59	—	—	12	78	2	8
12	Ag_2_H_1_PMo_12_O_40_^c^	DHA	35	70	45	—	—	31	64	1	4
13	Ag_1_H_2_PMo_12_O_40_^c^	DHA	27	62	35	—	—	40	56	1	3
14	H_3_PMo_12_O_40_^c^	DHA	13	40	17	—	—	54	42	1	3
15	Ag_3_PMo_12_O_40_^d^	GCA	19	52	24	—	—	42	46	9	3
16	Ag_2_H_1_PMo_12_O_40_^d^	GCA	16	48	20	—	—	45	42	10	3
17	Ag_1_H_2_PMo_12_O_40_^d^	GCA	12	40	15	—	—	50	38	10	2
18	H_3_PMo_12_O_40_^d^	GCA	7	28	9	—	—	56	32	10	2
19	Ag_3_PMo_12_O_40_^e^	LA	2	3	—	—	—	—	—	—	47
20	Ag_2_H_1_PMo_12_O_40_^e^	LA	4	5	—	—	—	—	—	—	51
21	Ag_1_H_2_PMo_12_O_40_^e^	LA	6	8	—	—	—	—	—	—	55
22	H_3_PMo_12_O_40_^e^	LA	9	18	—	—	—	—	—	—	60

^a^CON denotes conversion.

^b^TOF = (concentration of formed LA, mol L^−1^)/((amount used HPA, mol L^−1^) × (reaction time, h)). Reaction conditions: 5 mL of 10 wt% aqueous solution of glycerol, 0.023 mmol catalyst, 60 °C, 5 h, 5 bar O_2_, 800 rpm.

^c^DHA as the substrate with the same reaction except the time was 3 h.

^d^GCA as the substrate with the same reaction except the time was 3 h.

^e^LA as the substrate with the same reaction except the time was 2 h.
