# Supplementary material for: Designation of highly efficient catalysts for one pot conversion of glycerol to lactic acid
Source: Sci Rep. 2016 Jul 19;6:29840. doi: 10.1038/srep29840 (PMC4949431; doi:10.1038/srep29840)
Supplement: Supplementary Information [file srep29840-s1.doc]

**Supplementary Information to**

**Designation of highly efficient catalysts for one pot conversion of glycerol to lactic acid**

Meilin Tao^1^, Dan Zhang^1^, Hongyu Guan^1^, Guohui Huang^2^*, Xiaohong Wang^1^*

^1^Key Lab of Polyoxometalate Science of Ministry of Education, Northeast Normal University, Changchun 130024, P. R. China, E-mail: wangxh665@nenu.edu.cn, Fax: 0086-431-85099759

^2^School of Biology, Northeast Normal University, Changchun 130024, People’s Republic of China


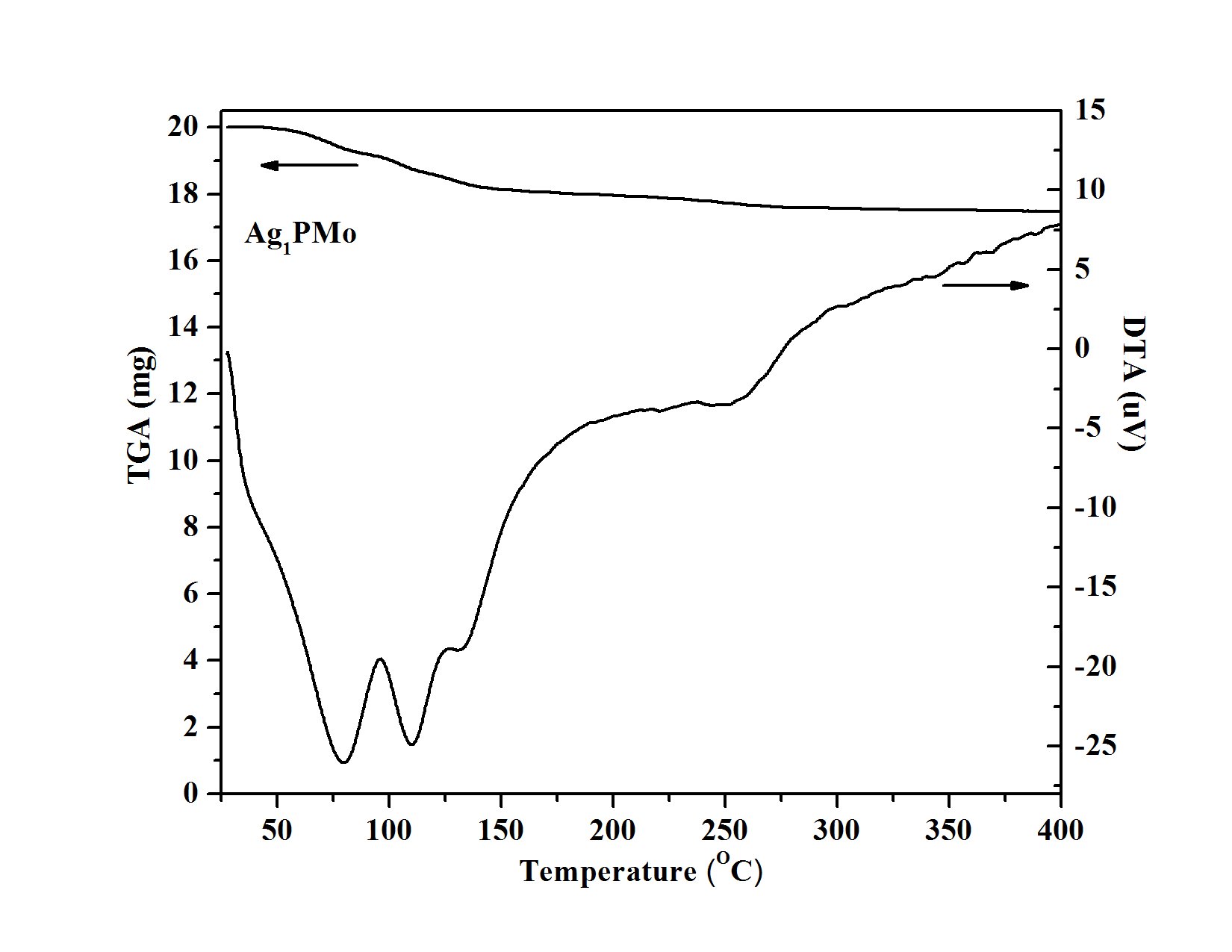

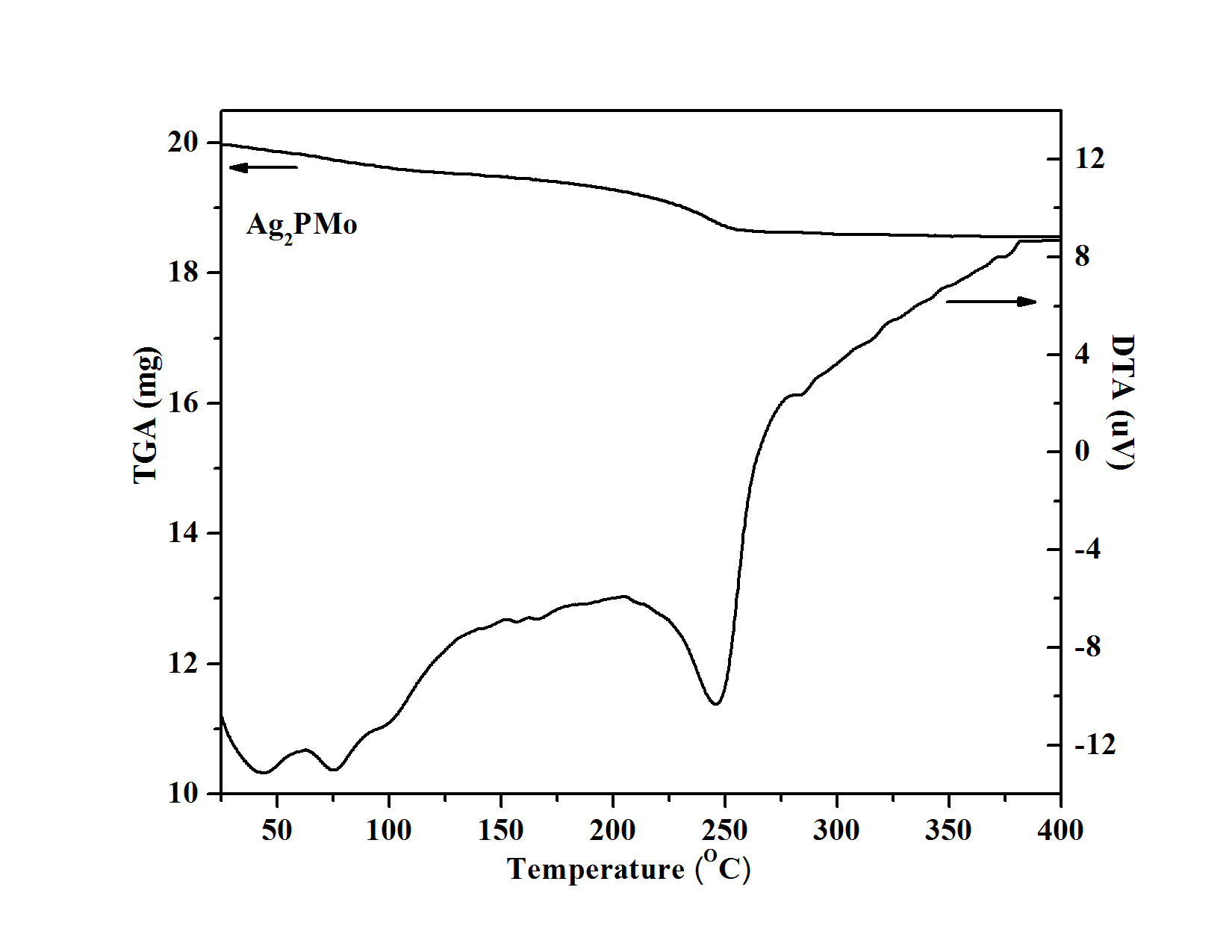

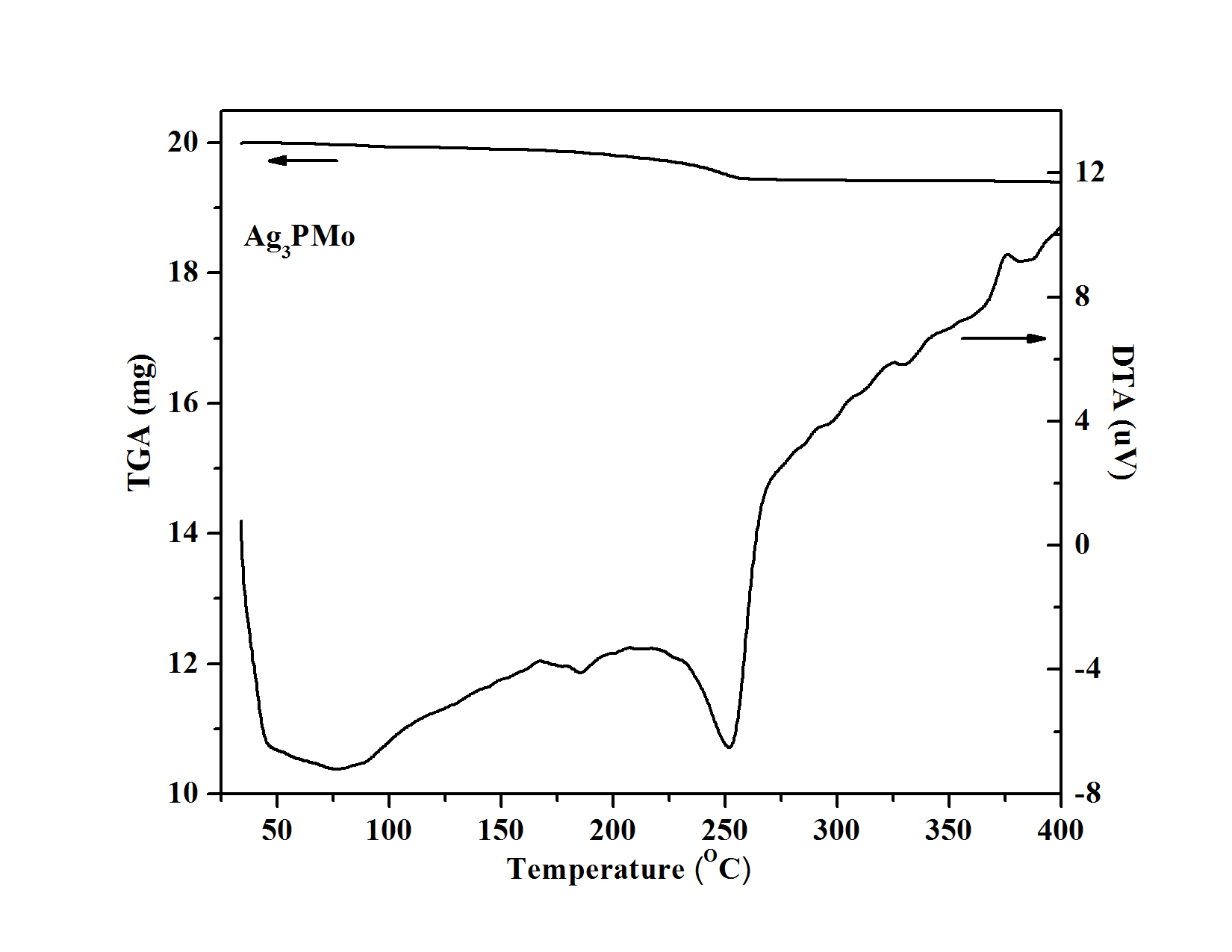


**Fig. S1.** TGA and DTA curves of Ag_X_PMo.

**xAg^+^**

**Exchanging**


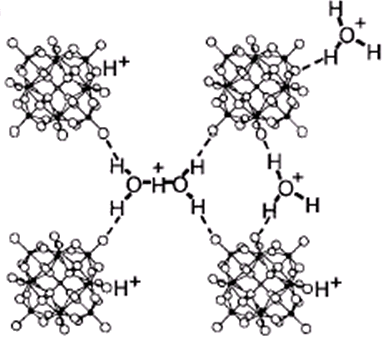

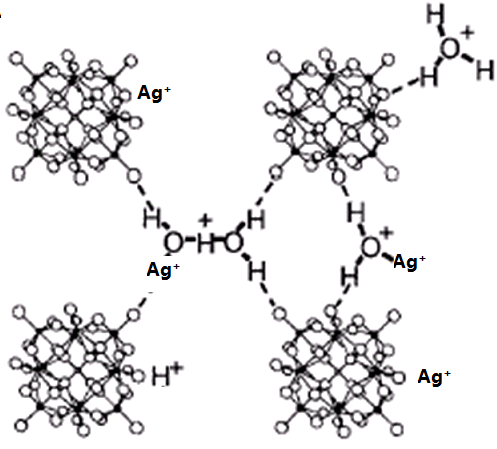


**Fig. S2.** The secondary structure of Ag_x_PMo as replacement of H^+^ (left, H_3_PM_o12_O_40_) by Ag^+^(right, Ag_x_PMo).


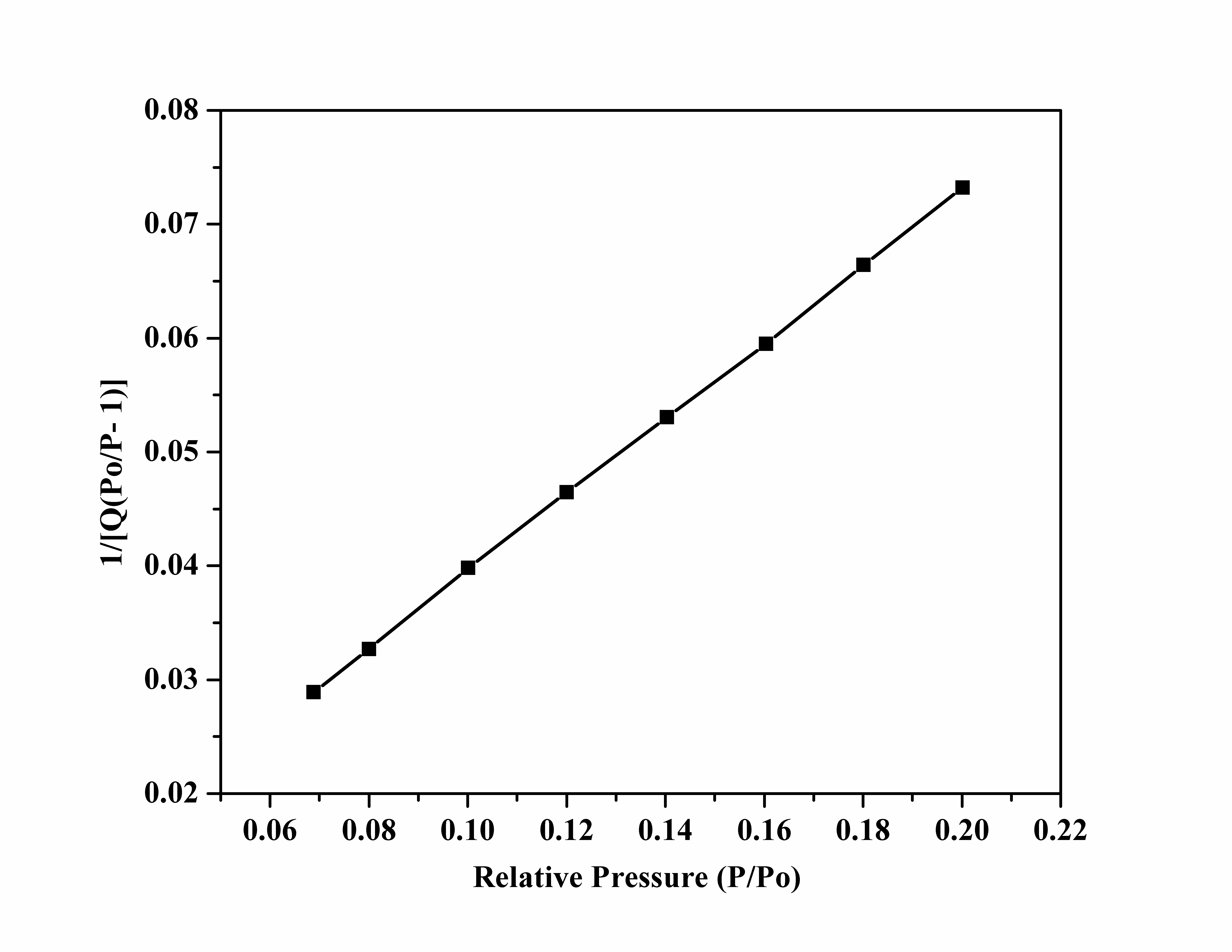

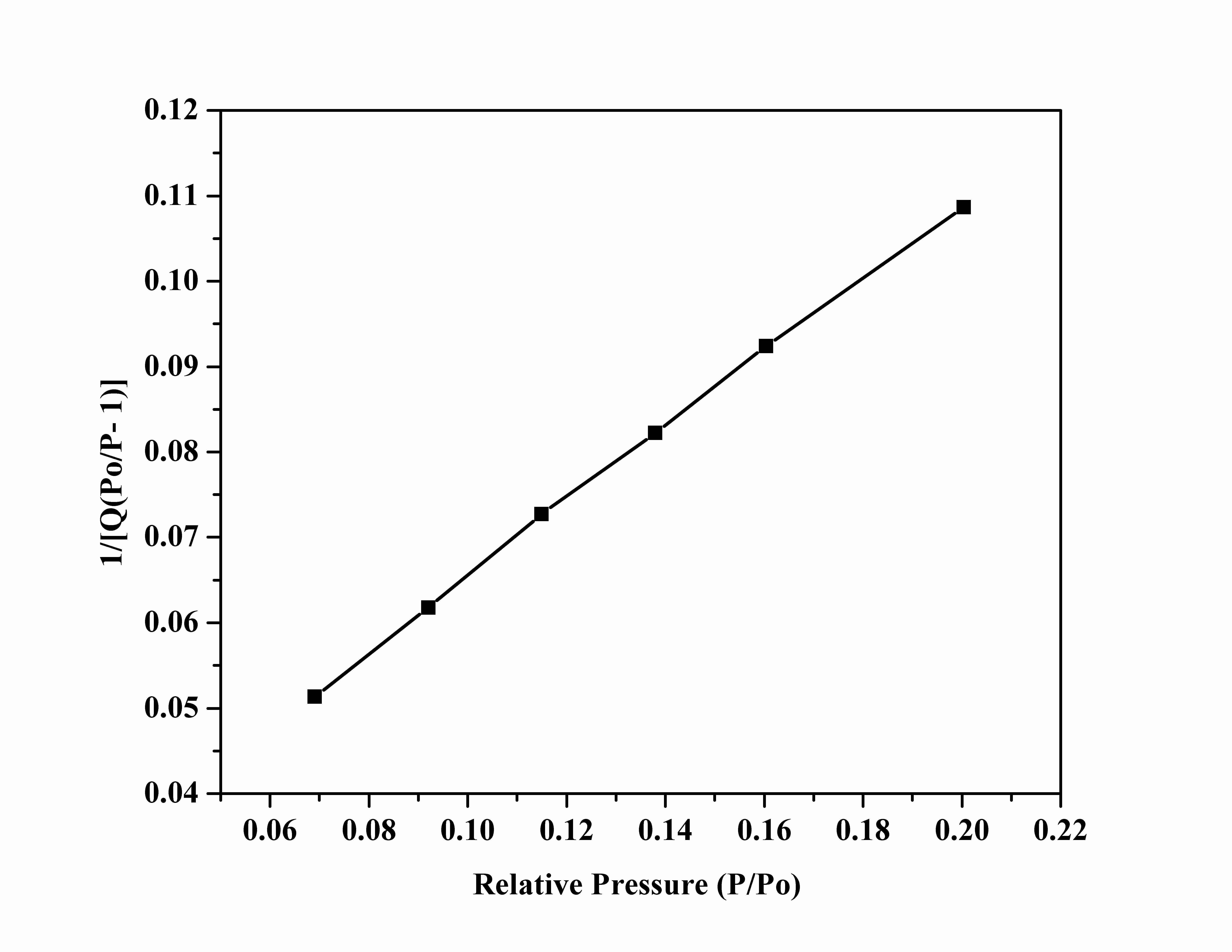

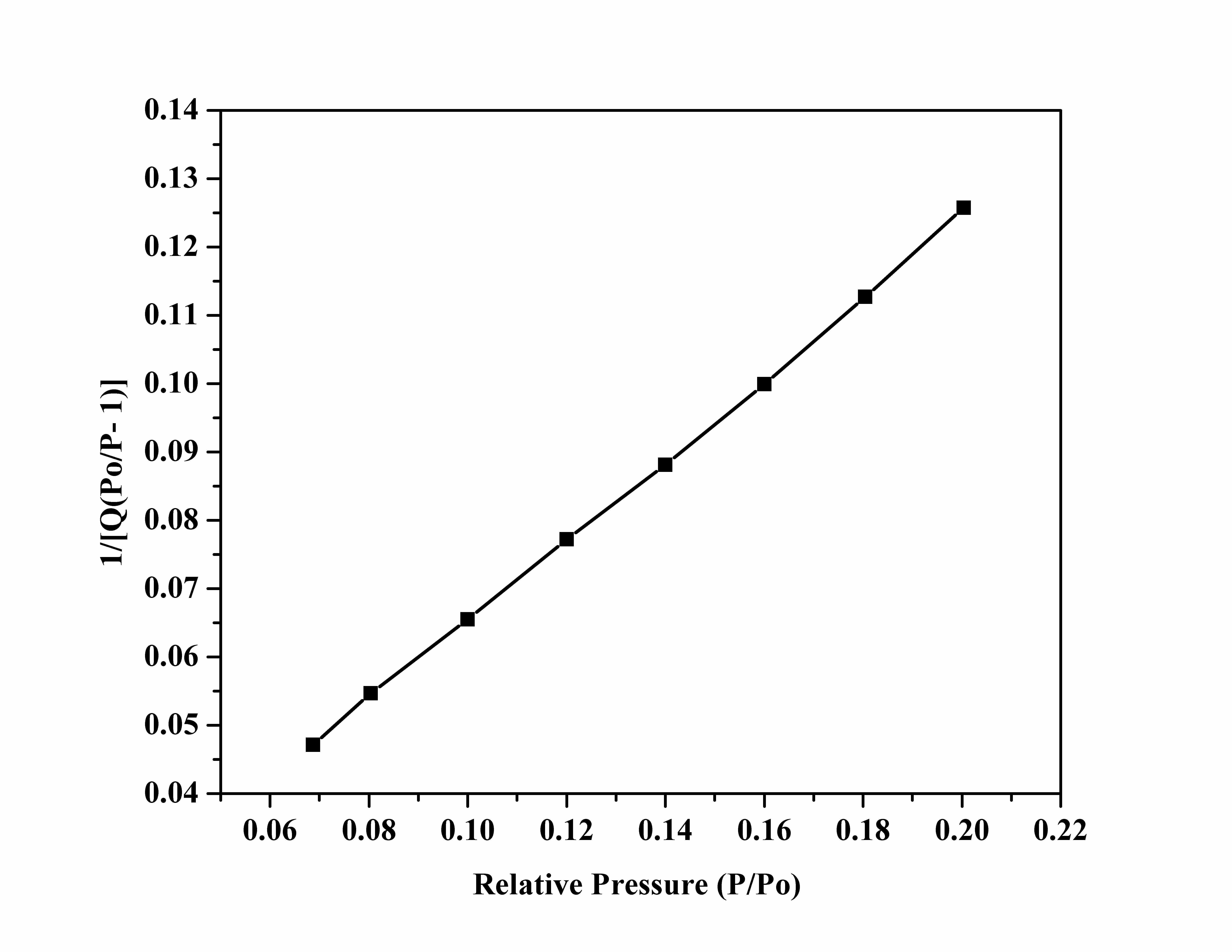


(d)

(e)

(f)


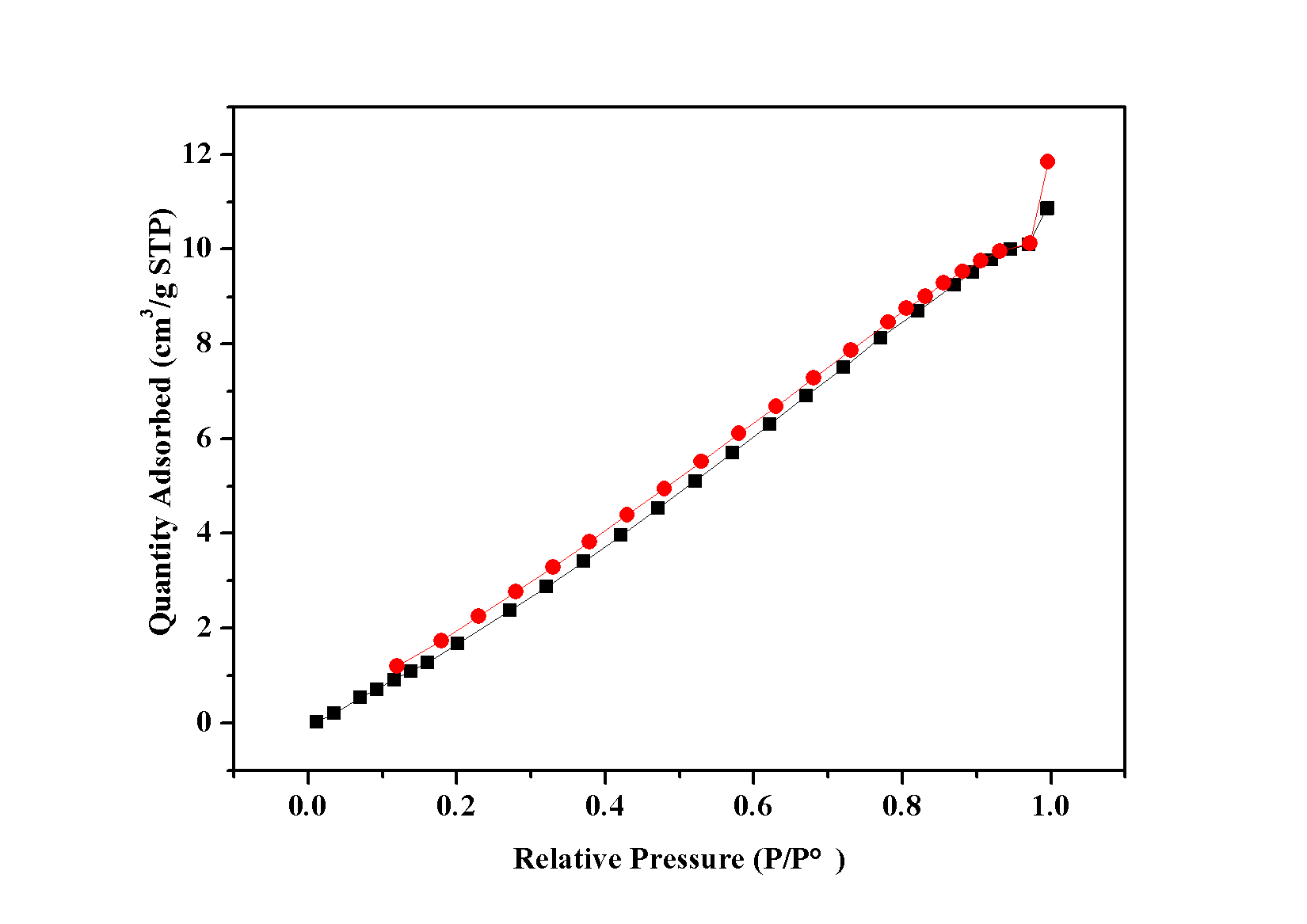

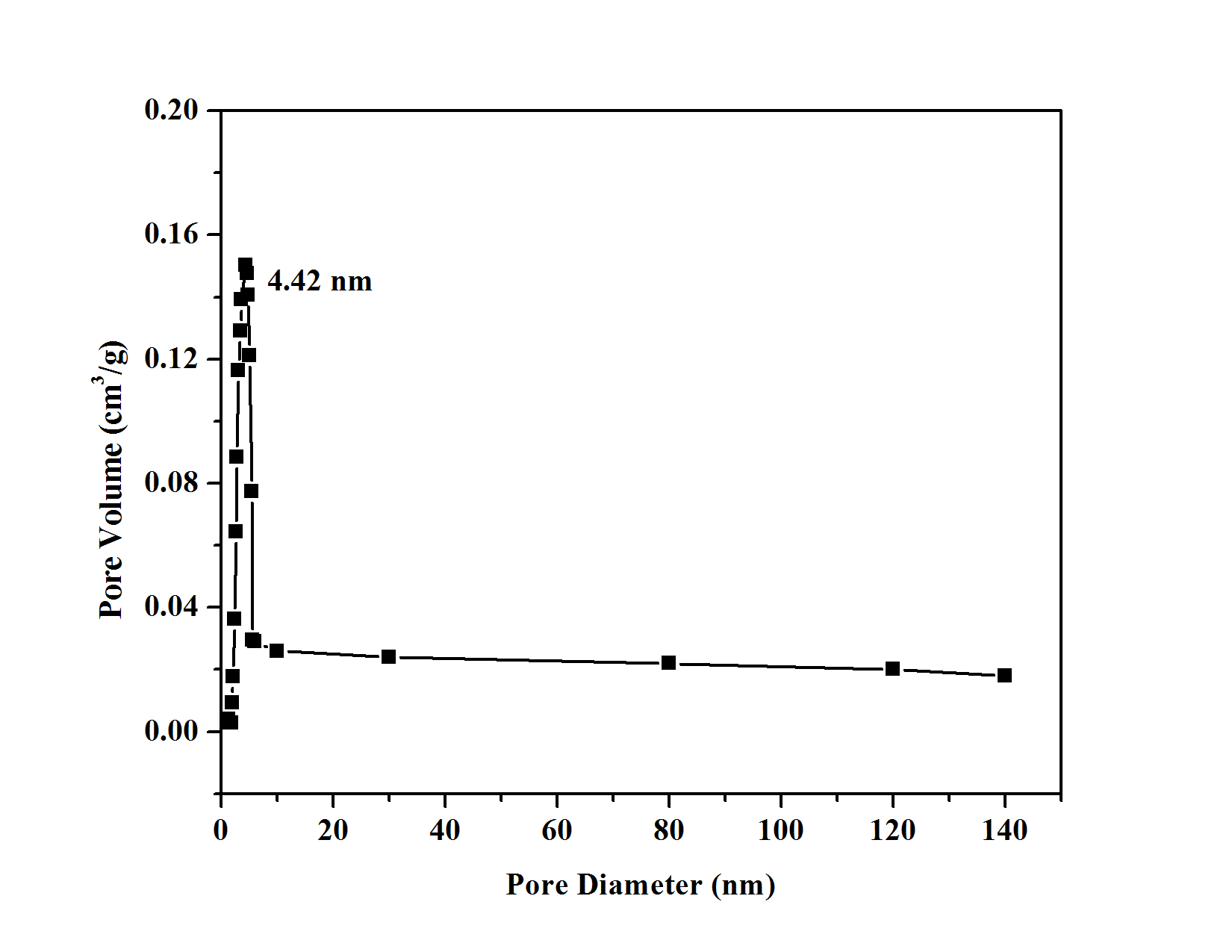


(a)


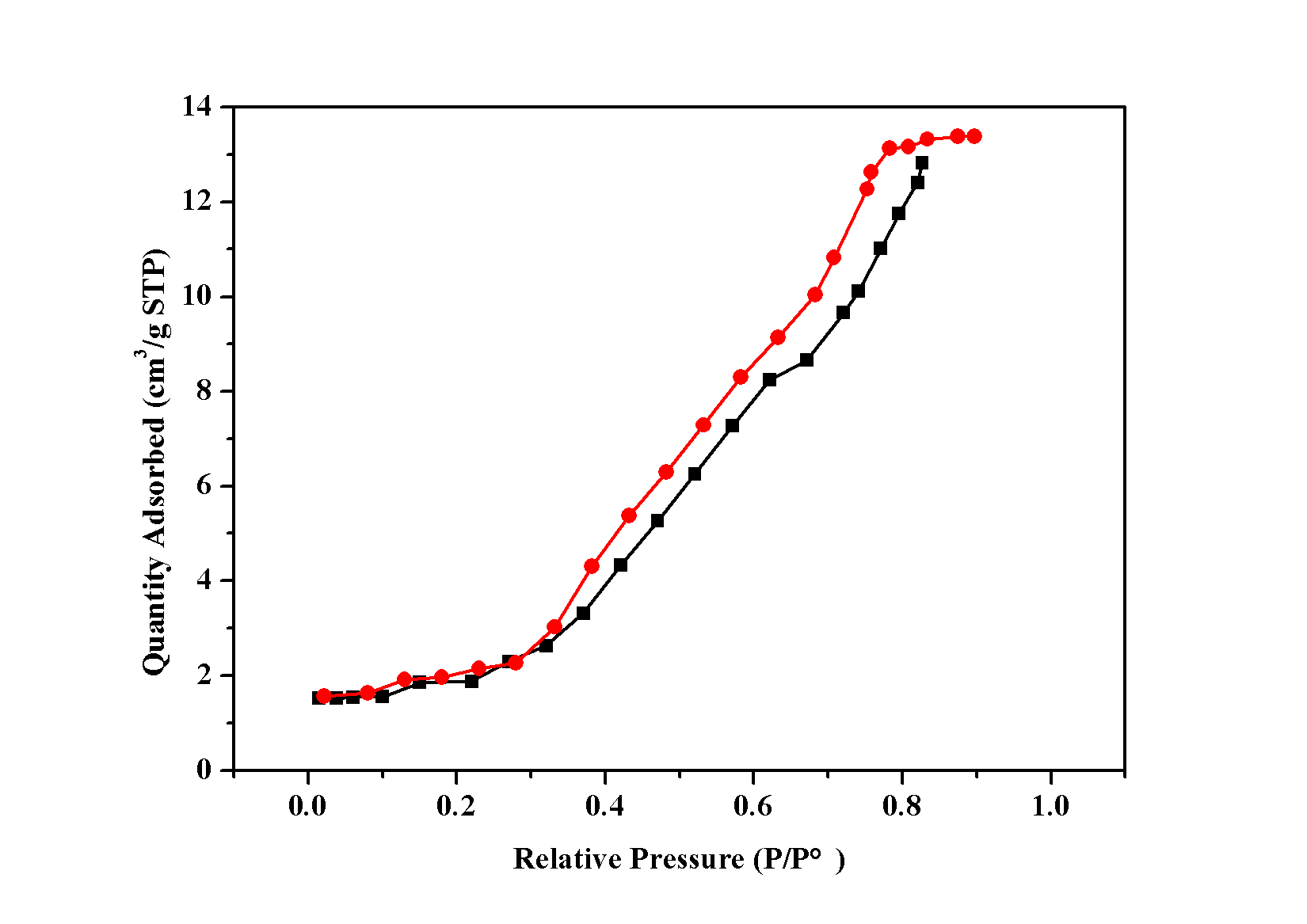

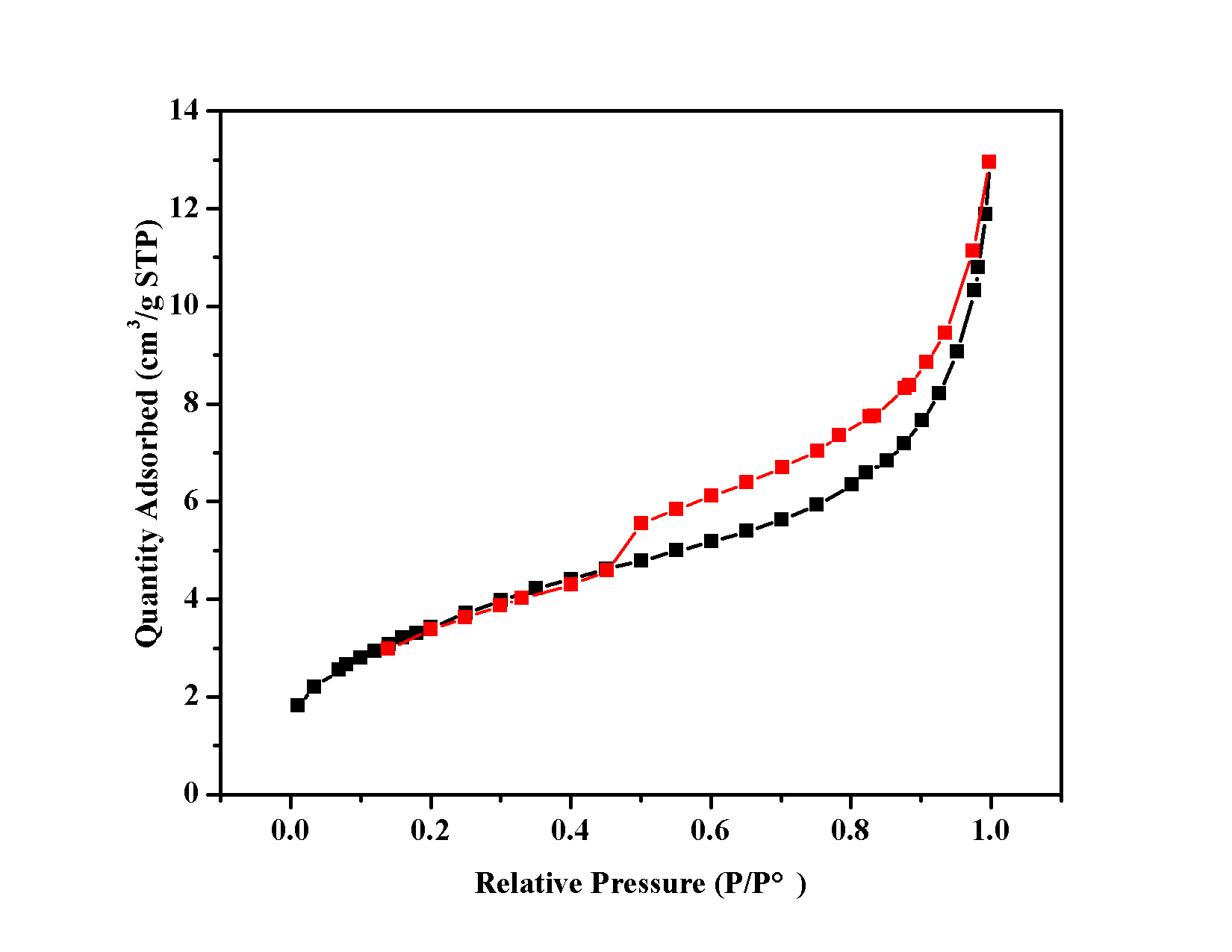

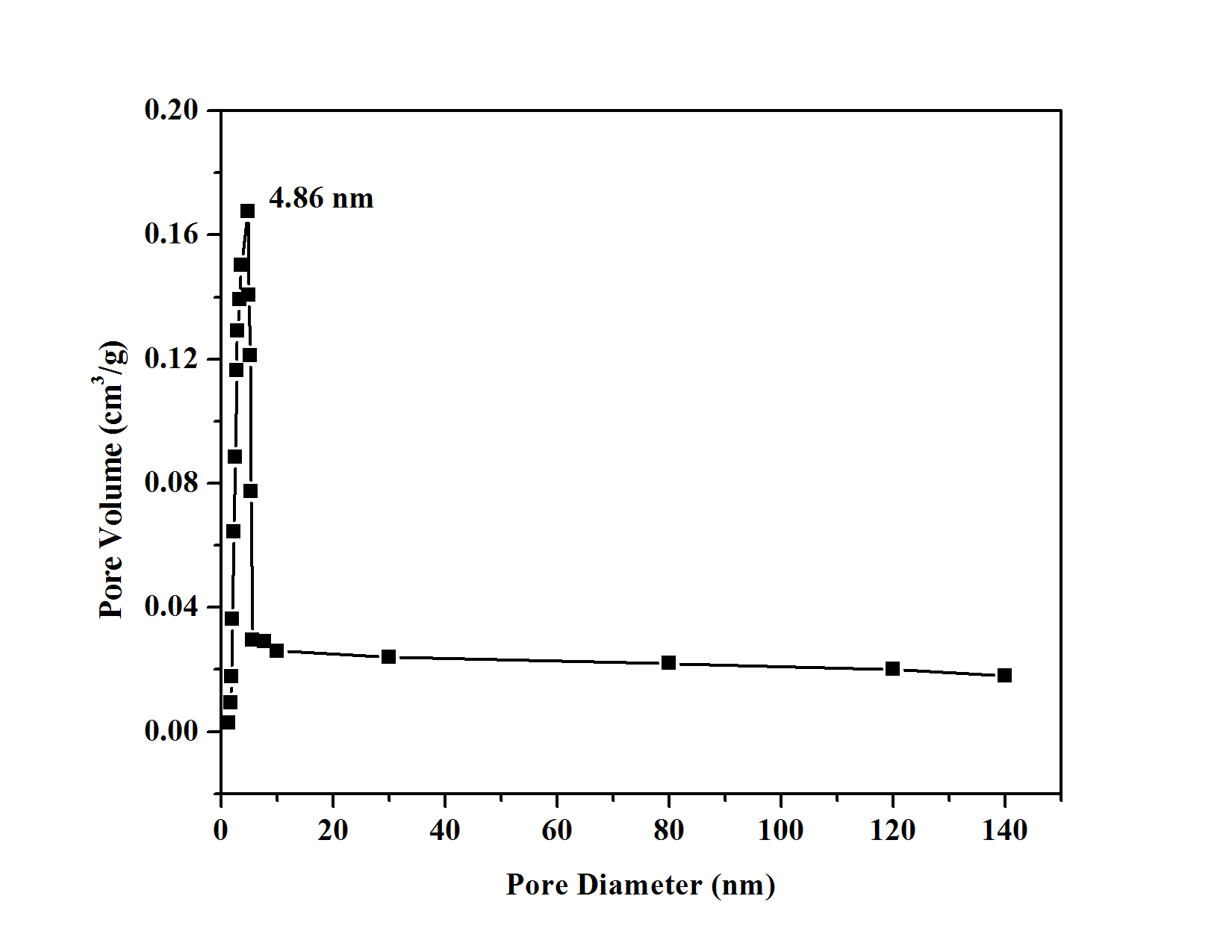

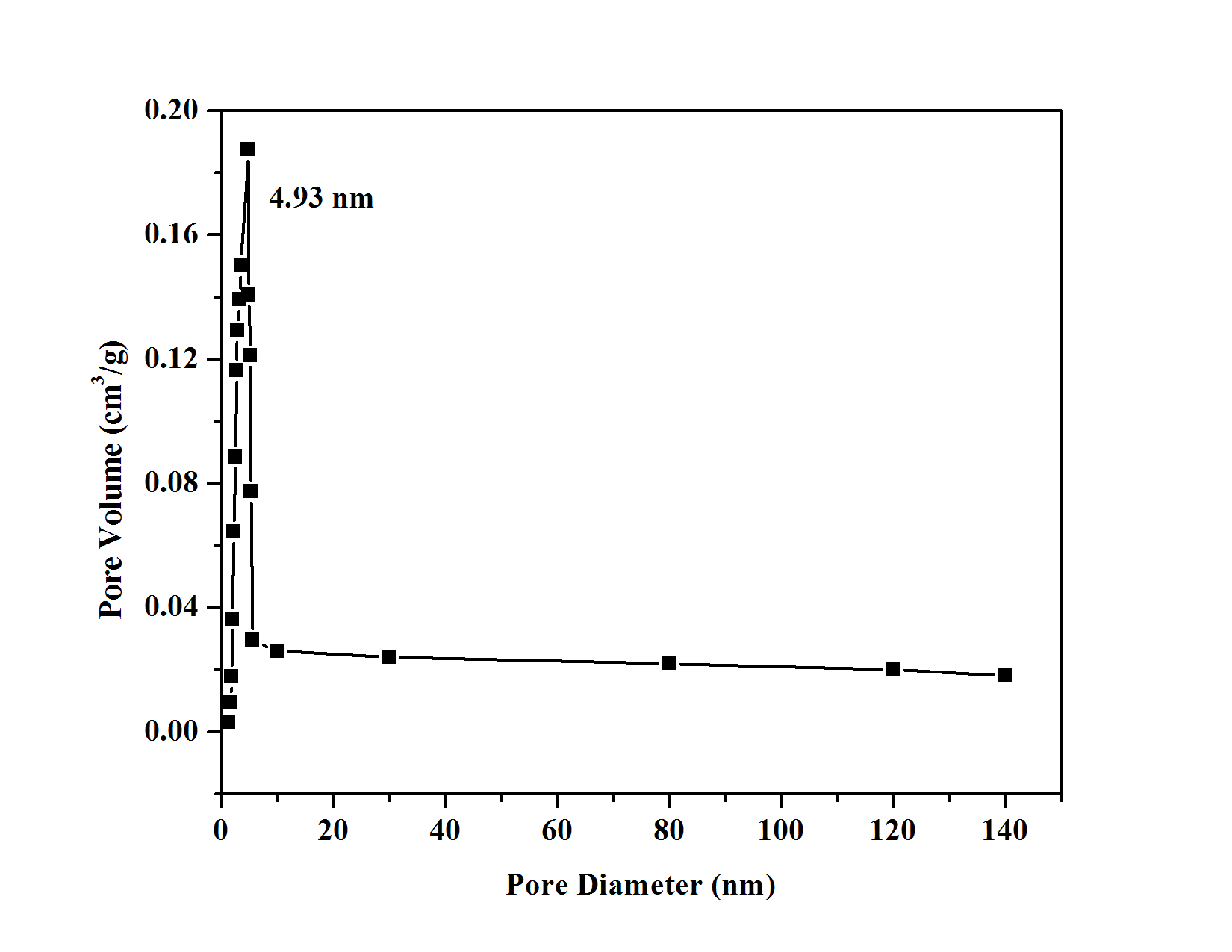


(b)

(c)

**Fig. S3.** N_2_ sorption isotherms and the pore size distribution of Ag_1_PMo (a), Ag_2_PMo (b) and Ag_3_PMo (c) and BET of Ag_1_PMo (d), Ag_2_PMo (e) and Ag_3_PMo (f).


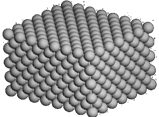

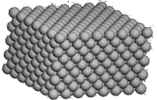

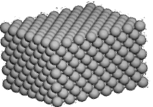

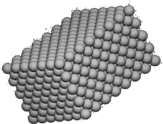

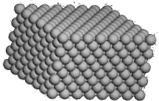


mesopores

micropores

micropores

micropores

**Fig. S4.** The formation mode of mesopores and micropores.

**
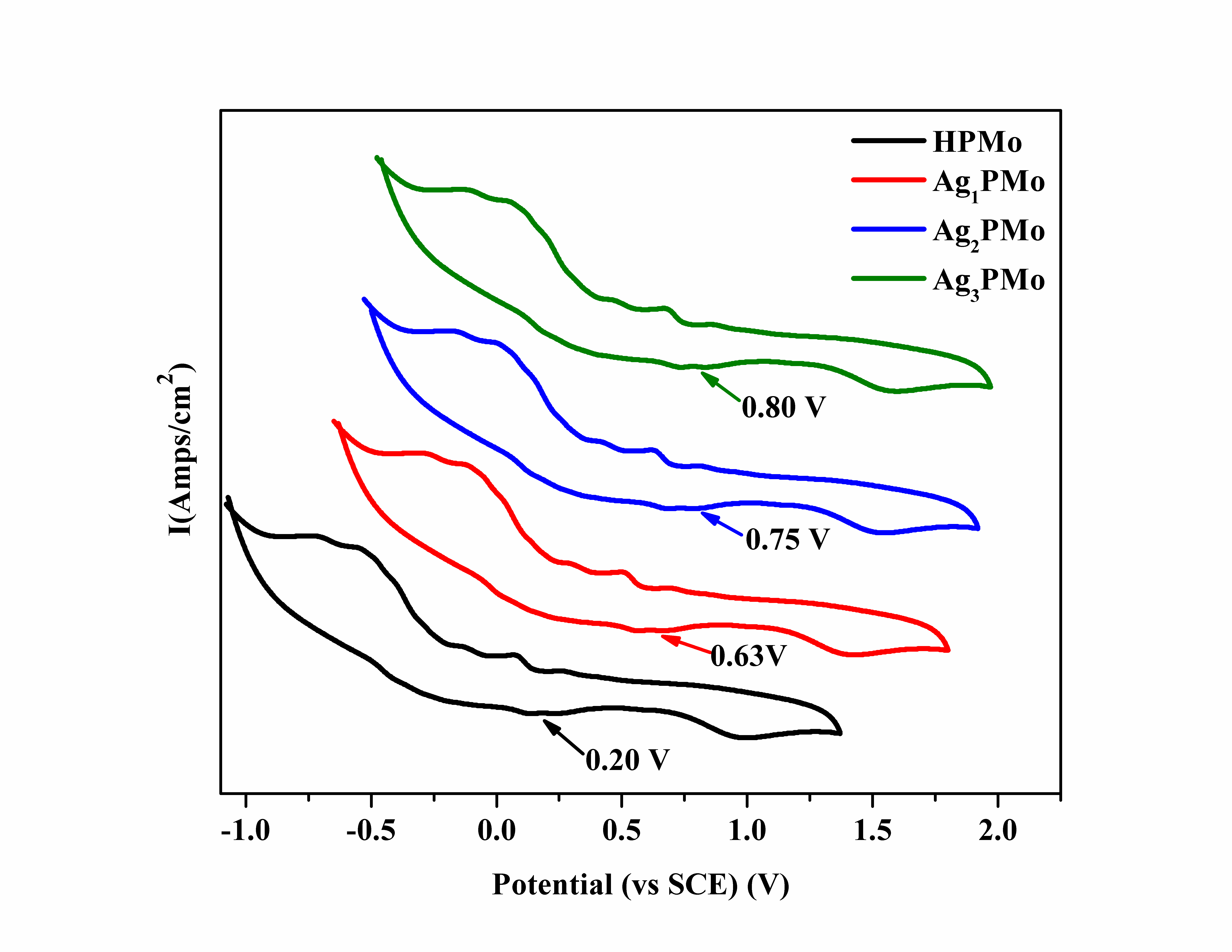
**

**Fig. S5**. CV-potential curves of Ag_x_PMo catalysts.


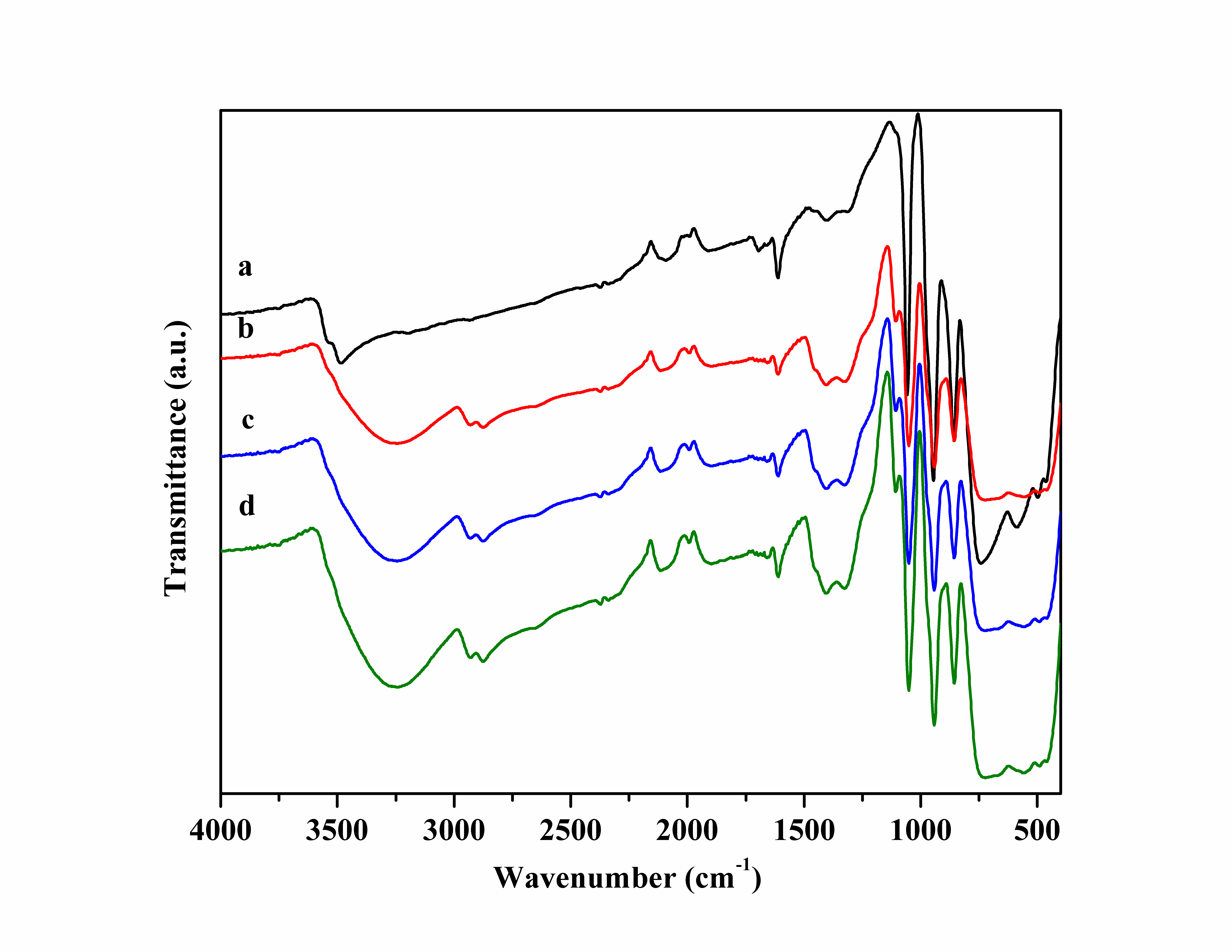


**Fig. S6.** FTIR spectra of Ag_3_PMo before (a) and after absorbing glycerol corresponding to Ag_1_PMo(b), Ag_2_PMo(c) and Ag_3_PMo(d), respectively.

**(e)**

**(c)**

**(a)**

**(d)**

**(b)**

**Fig. S7.** The conversion and selectivity of oxidation of glycerol with various process parameters:

(a) Reaction temperature (2.3×10^-5^ mol of Ag_3_PMo, 1.1 M of glycerol, 5 h,10 bar);

(b) Amount of catalyst (Ag_3_PMo, 1.1 M of glycerol, 60 ºC, 5 h, 10 bar);

(c) Reaction time (2.3×10^-5^ mol of Ag_3_PMo, 1.1 M of glycerol, 60 ºC, 10 bar);

(d) Concentration of glycerol (2.3×10^-5^ mol of Ag_3_PMo, 60 ºC, 5 h, 10 bar).

(e) The amount of oxygen (2.3×10^-5^ mol of Ag_3_PMo, 1.1 M of glycerol, 60 ºC, 5 h).





**Fig. S8.** Uv-Vis spectrum of the reaction mixture obtained after glycerol conversion over Ag_3_PMo.


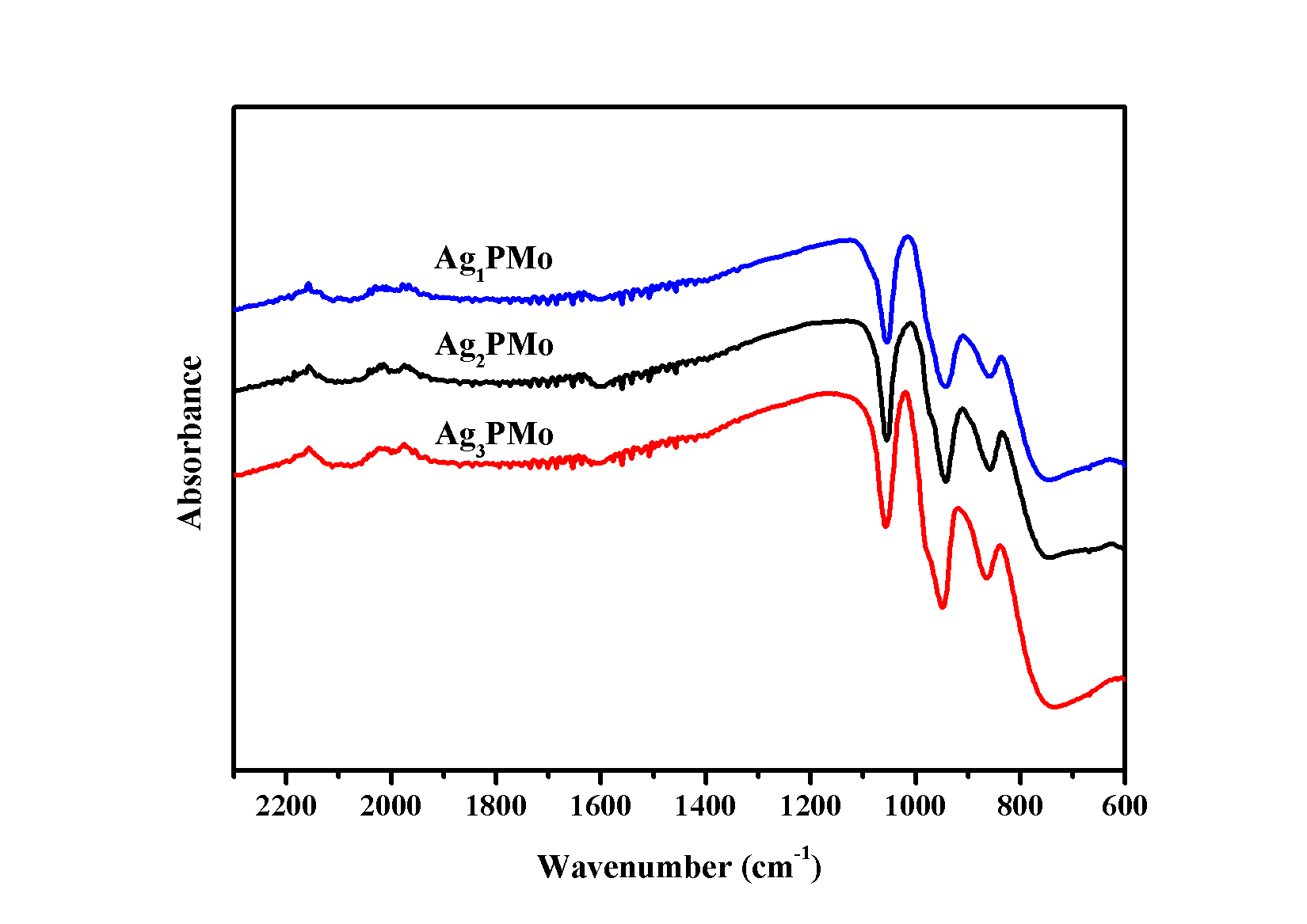


**Fig. S9.** FTIR spectra of catalysts after oxidation reaction.


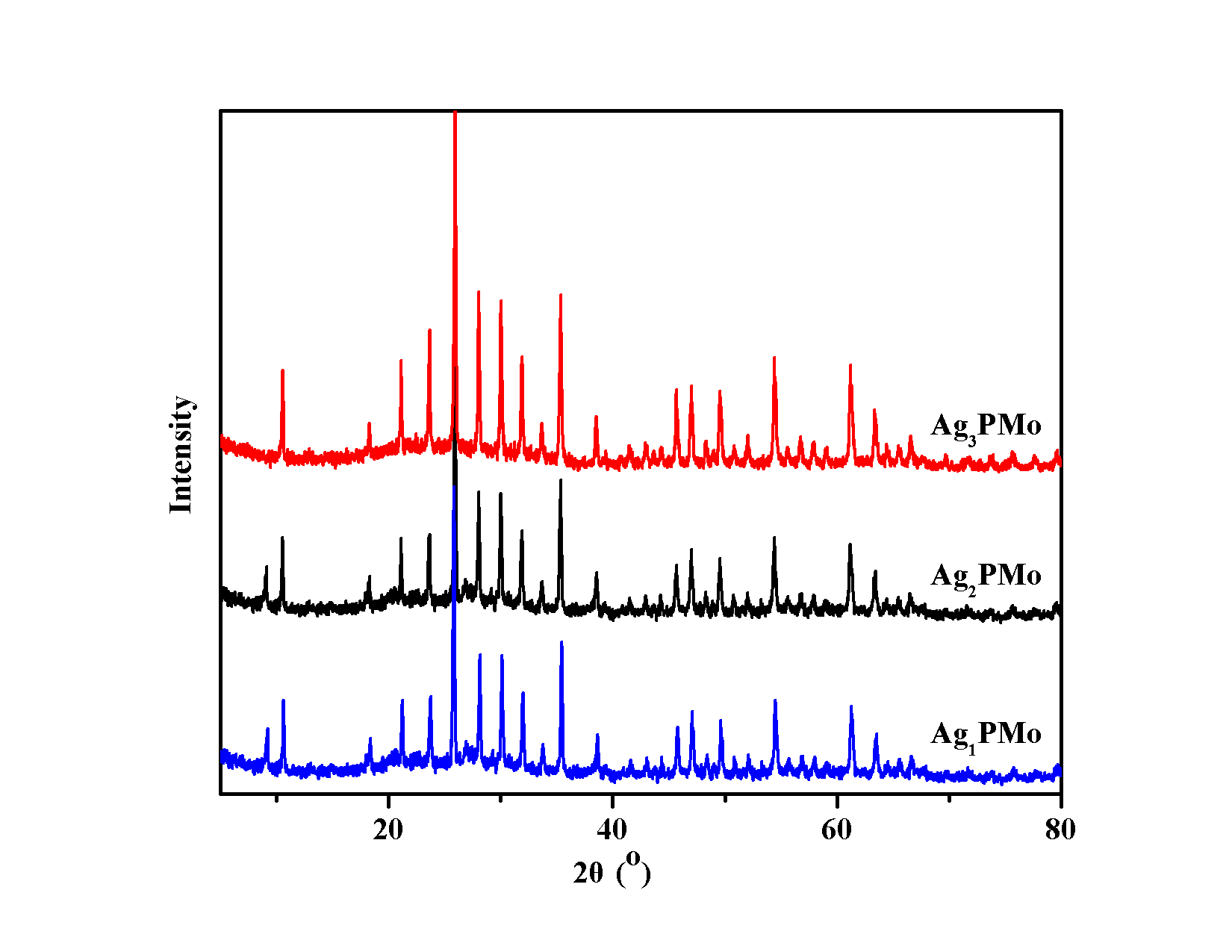


**Fig. S10.** XRD spectra of catalysts after oxidation reaction.


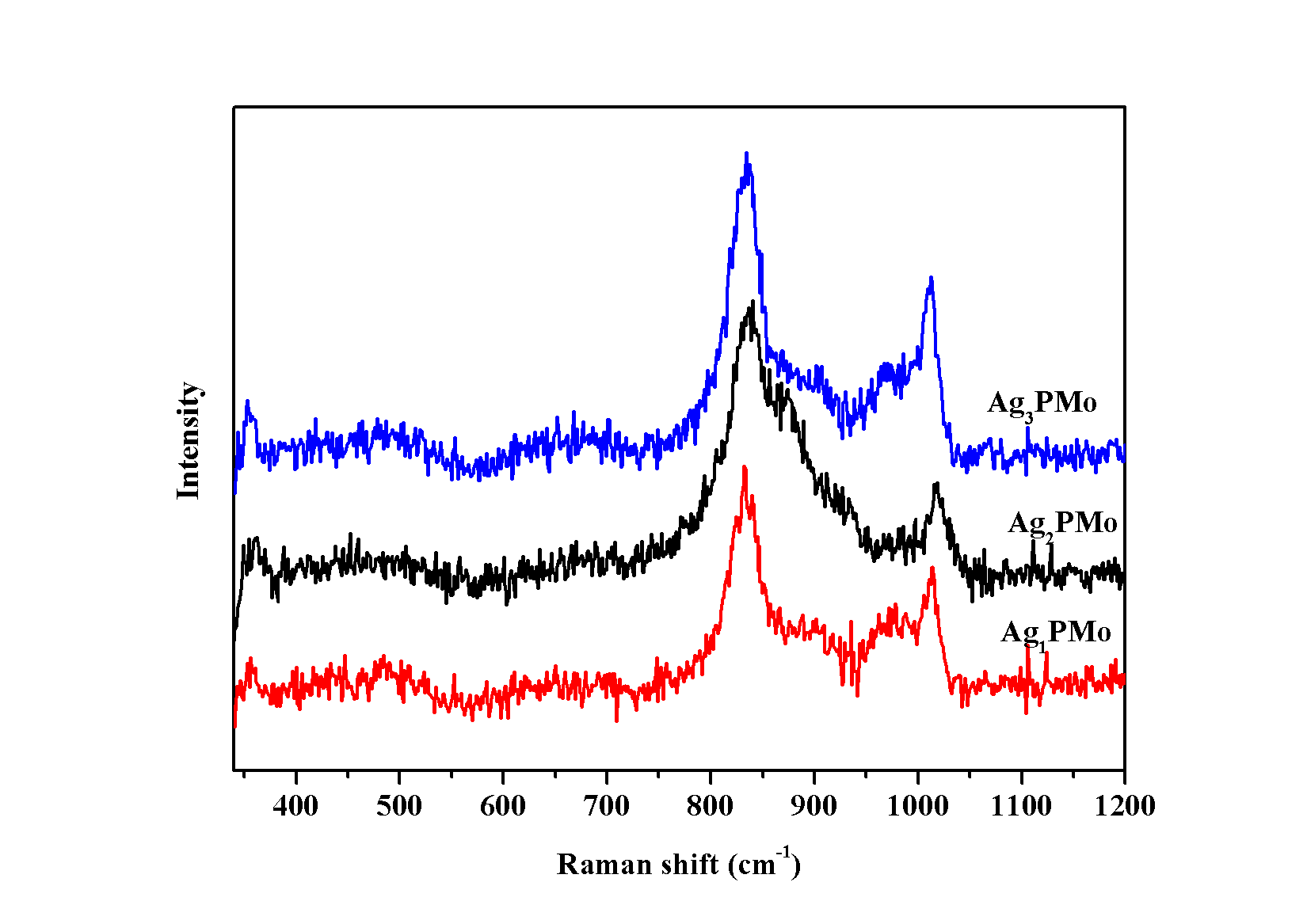


**Fig. S11.** Raman spectra of catalysts after oxidation reaction.


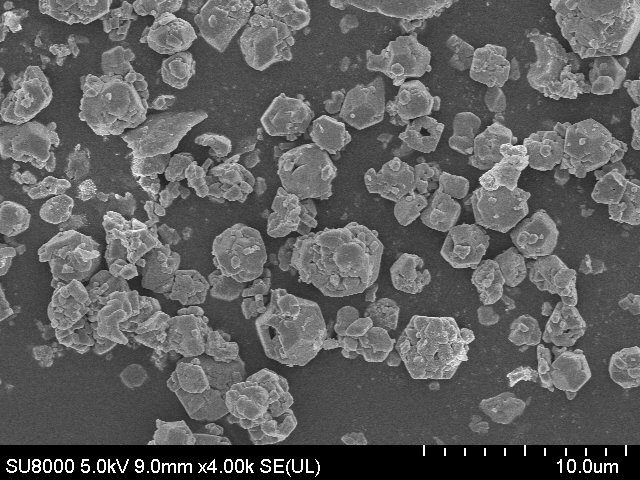

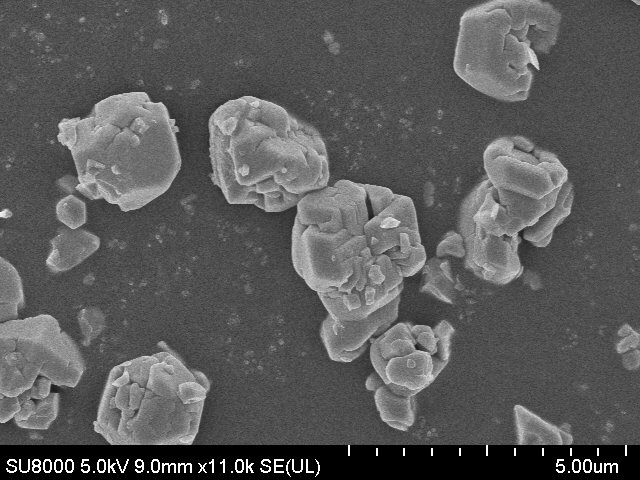

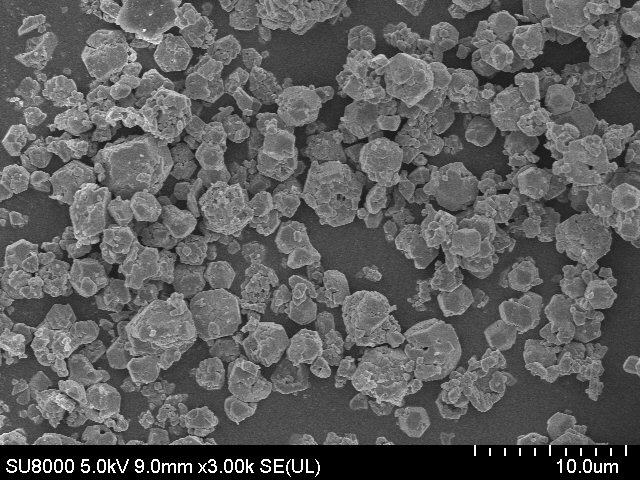


**b**

**c**

**a**

**Fig. S12.** SEM spectra of (a) Ag_1_PMo, (b) Ag_2_PMo, and (c) Ag_3_PMo after oxidation reaction.
